# Attenuation of TGFBR2 expression and tumour progression in prostate cancer involve diverse hypoxia-regulated pathways

**DOI:** 10.1186/s13046-018-0764-9

**Published:** 2018-04-27

**Authors:** Hui Zhou, Guanqing Wu, Xueyou Ma, Jun Xiao, Gan Yu, Chunguang Yang, Nan Xu, Bao Zhang, Jun Zhou, Zhangqun Ye, Zhihua Wang

**Affiliations:** 1 Department of Urology, Tongji Hospital, Tongji Medical College, Huazhong University of Science and Technology, Wuhan, 430030 China; 2 Institute of Urology, Tongji Hospital, Tongji Medical College, Huazhong University of Science and Technology, Wuhan, 430030 China; 3 Department of Urology, Aerospace Center Hospital(ASCH), Beijing, 100076 China; 4 Department of Urology, The third people Hospital of Hubei Province, Wuhan, 430030 China

**Keywords:** TGFBR2, Hypoxia, EZH2, MicroRNA-93, Prostate cancer

## Abstract

**Background:**

Dysregulation of transforming growth factor β (TGF-β) signaling and hypoxic microenvironment have respectively been reported to be involved in disease progression in malignancies of prostate. Emerging evidence indicates that downregulation of TGFBR2, a pivotal regulator of TGF-β signaling, may contribute to carcinogenesis and progression of prostate cancer (PCa). However, the biological function and regulatory mechanism of TGFBR2 in PCa remain poorly understood. In this study, we propose to investigate the crosstalk of hypoxia and TGF-β signaling and provide insight into the molecular mechanism underlying the regulatory pathways in PCa.

**Methods:**

Prostate cancer cell lines were cultured in hypoxia or normoxia to evaluate the effect of hypoxia on TGFBR2 expression. Methylation specific polymerase chain reaction (MSP) and demethylation agents was used to evaluate the methylation regulation of TGFBR2 promoter. Besides, silencing of EZH2 via specific siRNAs or chemical inhibitor was used to validate the regulatory effect of EZH2 on TGFBR2. Moreover, we conducted PCR, western blot, and luciferase assays which studied the relationship of miR-93 and TGFBR2 in PCa cell lines and specimens. We also detected the impacts of hypoxia on EZH2 and miR-93, and further examined the tumorigenic functions of miR-93 on proliferation and epithelial-mesenchymal transition via a series of experiments.

**Results:**

TGFBR2 expression was attenuated under hypoxia. Hypoxia-induced EZH2 promoted H3K27me3 which caused TGFBR2 promoter hypermethylation and contributed to its epigenetic silencing in PCa. Besides, miR-93 was significantly upregulated in PCa tissues and cell lines, and negatively correlated with the expression of TGFBR2. Ectopic expression of miR-93 promoted cell proliferation, migration and invasion in PCa, and its expression could also be induced by hypoxia. In addition, TGFBR2 was identified as a bona fide target of miR-93.

**Conclusions:**

Our findings elucidate diverse hypoxia-regulated pathways including EZH2-mediated hypermethylation and miR-93-induced silencing contribute to attenuation of TGFBR2 expression and promote cancer progression in prostate cancer.

**Electronic supplementary material:**

The online version of this article (10.1186/s13046-018-0764-9) contains supplementary material, which is available to authorized users.

## Background

Prostate cancer (PCa) is the most common malignant tumor in males with increasing incidence worldwide, causing immense mortality and heavy health burdens [1]. The number of newly diagnosed PCa is estimated to reach 160,000 men in the world, with existing 3.3 million survivors [2]. Although most of prostate cancers exhibit indolent courses, some tumors can also become metastatic and castration-resistant which ultimately cause cancer death in men. Active surveillance, surgical resection or ablation, and radiation continue to be recommended approaches for localized diseases, while androgen deprivation therapy, chemotherapy, and other targeted agents are utilized to extend survival in patients with metastatic diseases [3]. With the advances in surgical techniques, radiotherapy and hormone therapy, the barriers of PCa management becomes the methods to treat metastatic diseases, especially for the castration-resistant prostate cancer (CRPC) [4, 5]. Although plenty of new advances in genetics have already deepened our understanding of this disease beyond androgen receptor (AR) pathways [6, 7], the precise regulatory mechanisms of PCa metastasis are still not fully elucidated, with scarcity of effective therapeutic targets and corresponding clinical targeted agents. As a result, it is of paramount importance to further elucidate the cellular and molecular mechanisms involved in PCa for developing anticancer therapies.

Epithelial-mesenchymal transition (EMT) is a multifunctional biological process required for embryonic development, wound healing, and tissue reconstruction, while aberrant EMT engages in some pathologic processes such as tumor progression, invasion, and metastasis [8]. During EMT, cells undergo morphologic changes from well differentiated epithelial phenotype to invasive mesenchymal one, which promotes metastasis and therapeutic resistance. EMT also impacts on the development and progression of metastatic PCa, and therapeutic targeting of EMT has the potential to shift the treatment paradigm of CRPC [9]. Bone is the most common metastatic lesion in prostate cancer patient, while transforming growth factor β (TGF-β) signaling is identified to affect the metastatic tumor cell proliferation and osteolytic bone metastasis in several cancer types [10]. As a transmembrane serine-threonine kinase, type II TGF-β receptors (TGFBR2) is a major member to initiate downstream TGF-β signaling in tumorigenesis. The expression of TGFBR2 is often found to be altered in several types of malignance, such as metastatic breast cancer [11], colorectal cancer [12], and prostate cancer [13]. Loss of TGFBR2 expression in majority of human PCa-associated stoma was observed, which might relieve the paracrine suppression of Wnt3a and promote the progression of PCa [13]. Besides, inactivation of TGF-β pathway by deleting TGFBR2 in combination of APC tumor suppressor deletion could result in rapid onset of invasive PCa, which implied TGFBR2 might exert tumor suppressive effect in prostate [14]. Methylation silencing of TGFBR2 was found in rat prostate cancers [15], further studies also proved AR/miRNA-21 or AR/miR-2909 positive feedback loop down-regulated the expression of TGFBR2, leading to attenuation of TGF-β mediated Smad2/3 activation and subsequent tumor-suppressive activity [16, 17]. These results demonstrate the critical roles of TGFBR2 in the progression of PCa and its down-regulation in PCa by promoter methylation and certain miRNAs, while the inquiry of more detailed mechanisms regarding to TGFBR2 in PCa is still needed.

It has been identified that the degree of hypoxia in prostate cancer positively correlates with cancer progression and worse prognosis. Hypoxia-inducible factor 1α (HIF-1α) expression is also shown to increase in PCa tissues. Increased expression of intrinsic markers of tumor hypoxia and angiogenesis such as VEGF, HIF-1α, and osteopotin indicate higher risk of biochemical failure [18]. Moreover, nonspecific HIF-1α inhibitors are shown to increase the progression-free survival (PFS) and reduce the risk of CRPC and metastases [19]. It is clear that hypoxia-regulated molecular events can exert important effects on the development of PCa and CRPC, and targeting the hypoxia signaling has the potential to confront this thorny malignance. Mechanistic investigation has found out that hypoxia-induced PHF8 can serve as an essential histone demethylase activity-dependent AR coactivator, promoting AR signaling pathway and cancer progression [20]. Hypoxia can also regulate mTOR signaling which is critical for PCa stem cell survival [21]. Besides, several miRNAs such as miR-182 and miR-101 can be included by hypoxia in prostate cancer [22], epigenetic modulators such as Enhancer of zeste homolog 2 (EZH2) can also be regulated by HIF-1α induction [23]. However, the actual effect and precise signaling pathways of hypoxia in prostate cancer are still largely elusive at the moment, more downstream effector molecules and associated pathways are in urgent need to be identified. Our experiments found out that hypoxia could attenuate the expression of TGFBR2, whereas the underlying mechanism and significance of this regulation in prostate cancer remain poorly understood.

In this study, we demonstrated the impact of hypoxia on the expression of TGFBR2 in prostate cancer cells. Furthermore, several hypoxia-regulated molecules and signalings such as miRNAs and epigenetic regulators were explored to resolve the mechanism of TGFBR2 down-regulation by hypoxia. Finally, we inquired into the molecules involved in the regulatory network for their potential of biomarkers and therapeutic targets in PCa.

## Methods

### Patients and tissue specimens

Paired PCa and adjacent normal tissue samples (collected postoperatively from September 2010 to February 2017) were obtained from 56 patients who were admitted to and underwent PCa resection in the Department of Urology, Tongji Hospital, Huazhong University of Science and Technology (Wuhan, China). Specimens were obtained under the informed consent of each patient and the approval of Ethics Committee of Tongji Hospital. All the diagnoses were validated by pathology reports. On removal of the surgical specimen, each sample was immediately frozen in liquid nitrogen and stored at − 80 °C until use. The clinicopathologic characteristics of patients are summarized in Additional file 1: Table S1.

### Cell culture and transfection

Human prostate cancer cell lines (PC3, DU145 and LNCap) were obtained from the American Type Culture Collection (Manassas, VA), and a primary culture of human RWPE-1 was obtained from the Shanghai cell bank of the Chinese Academy of Sciences. Generally, PC3, DU145 and LNCap were cultured in RPMI-1640, while RWPE-1 was culture in K-SFM medium, supplemented with 10% fetal bovine serum (FBS) and placed in incubator with 5% CO2 at 37 °C. For hypoxic conditions, cells were cultured in a Forma Series II Water Jacket incubator with 1% O2, 5% CO2 and 94% N2 (Thermo Scientific).

Synthetic, chemically modified short single or double stranded RNA oligonucleotides and corresponding negative control were purchased from Ribo Biotech (Guangzhou, China). SiRNA sequences are available in Additional file 1: Table S2. For transfection, cells were seeded into 6-wells plate and incubated overnight, and then 100 nM of small RNA molecules were transfected into cells by using X-tremeGENE siRNA transfection reagent (Roche). Besides, plasmids were transfected into cells with X-tremeGENE HP DNA Transfection reagent (Roche).

### RNA extraction and real-time polymerase chain reaction analysis

Total RNA from tissues and cultured cells were isolated using TRIzol reagent (Invitrogen, USA). Total RNA measuring 1000 ng was reverse transcribed, and real-time quantitative polymerase chain reaction (RT-qPCR) was performed on the Mx3000P system (Stratagene, USA) according to the manufacturer’s instructions. A qRT-PCR analysis was performed with the Platinum SYBR Green qPCR SuperMix kit (Invitrogen, Carlsbad, CA). U6 snRNA and GAPDH were used as internal controls for miR-93 and TGFBR2, respectively. All primers sequences used are available in Additional file 1: Table S2.

### Protein isolation and western blot analysis

Whole-cell lysates were prepared with RIPA lysis buffer of appropriate volume. Proteins were separated by 10% SDS-PAGE gel and transferred to PVDF membrane, following membrane blocking with 5% Albumin from bovine serum (BSA). Then the membranes were incubated in primary antibodies against targeted protein at 4 °C overnight, and after further incubated with HRP-conjugated secondary antibody, the bands were scanned. Antibodies used in experiments included: anti-TGFBR2 (Cell Signaling Technology, USA), anti-EZH2 (Abcam, USA), anti–HIF1A (Cell Signaling Technology, USA), anti-HIF2A (Cell Signaling Technology, USA), and anti-H3K27me3 (Abcam, USA). All primary antibodies except for internal control were used at 1:1000 dilution. GAPDH and α-tubulin (Boster, China) were used as the internal control at 1:5000 dilution.

### Demethylation assay and methylation-specific polymerase chain reaction (MSP)

To determine whether TGFBR2 expression can be elevated in prostate cancer cells by demethylation, we treated the cells with 5-aza-2′-deoxycytidine (5-Aza-DC). Cells were seeded in 6-well plates and treated with 5-Aza-DC (Sigma-Aldrich) at the final concentration of 10 μM, and culture for 72 h before the total RNA was extracted. The relative expression of TGFBR2 was measured by qRT-PCR with the untreated cells as control.

Genomic DNA from prostate cancer tissues and corresponding normal tissues and prostate cell lines was isolated using a simplified Proteinase K digestion method, and then bisulfited using Epitect Fast Bisulfite Conversion Kit(Qiagen) according to the manufacturer’s instructions. Then the methylation status of TGFBR2 promoter was determined by methylation specific PCR by amplified the genomic DNA after standard sodium bisulfate modification. Primer sequences which specifically recognized either the methylated or unmethylated TGFBR2 promoter were designed with Methprimer (http://www.urogene.org/cgi-bin/methprimer/methprimer.cgi). The PCR conditions were: 95 °C for 5 min, followed by 40 cycles of 95 °C for 15 s, 57 °C for 30s, 72 °C for 30s; and a final extension at 72 °C for 10 min. PCR products were separated on 1.5% agarose gels which stained with Gel-Red, and visualized under UV illumination.

### Cell proliferation and viability assay

Proliferation of cells was detected by the MTS assay using the CellTiter 96®AQueous One Solution Cell Proliferation Assay kit (Promega). One thousand treated cells were seeded in each well in a 96-well plate, the absorbance at 490 nm was measured every 24 h from day 0 to 4.

For colony formation assay, 1000 treated cells were seeded into 6-wells plates and cultured for another 10–14 days. The colonies were stained with 0.5% crystal violet (Sigma, USA) and counted.

### Flow cytometry for apoptosis

Cell apoptosis was detected via Annexin V Apoptosis Detection Kit APC (eBioscience, Affymetric). Briefly, the cells were washed in PBS and resuspended in 1 × binding buffer. Then the cells were stained by fluorochrome-conjugated Annexin V for 15 min at room temperature. After we washed the cells and resuspended them in binding buffer, we stained the cells with Propidium iodide (PI) staining solution and then analyzed them by flow cytometry.

### Transwell assay for migration and invasion

About 5 × 10^4^ of PC3 and DU145 cells were plated in the upper chambers of 24-well Transwell plates (Corning) in FBS-free medium. Complete medium (10% FBS) was deposited in the lower chambers to serve as a chemo-attractant. After 12 h, cells remaining on the upper filter were removed, while cells that passed through the Transwell filter were stained by 0.5% crystal violet for 20 min. Images were taken of five random optical fields (200×) on each filter and cell number was quantified by utilizing the ImageJ software (National Institutes of Health, USA).

To evaluate cell invasion, Transwell membranes were coated with Matrigel (BD Biosciences) prior to seeding treated cells or control cells. The Matrigel served as a basement membrane barrier that cells would have to destroy in order to invade the lower chamber. After 24 h for PC3 and DU145, crystal violet staining and cell counting were performed as above.

### Luciferase reporter assays

After constructing the wild-type 3′-untranslated region (3’-UTR) of TGFBR2 using PCR, we sub-cloned it into the NotI and XhoI sites in the luciferase reporter plasmid psiCHECK2 (Promega). All used primers were included in Additional file 1: Table S3. DNA sequencing was used to validate the validity of plasmids. After co-transfection with siRNAs and plasmids, cells were cultured for another 48 h before harvested. Then the cells were lysed and assayed for Renilla and Firefly luciferase activity using Dual-Luciferase Reporter Assay System (Promega). The ratio of Renilla luciferase intensity to luciferase one was calculated for each assay.

### Statistical analysis

Data were expressed as mean ± standard deviation, the Student t-test was used to analyze data between two groups, and one-way ANOVA was used to assess the multiple comparisons between groups. Correlation between the expression of two molecules was analyzed using the Pearson correlation coefficient analysis. All statistical analyses were performed using SPSS 21.0 (SPSS, Chicago). *P* values < 0.05 were considered to be statistically significant.

## Results

### TGFBR2 expression was decreased under hypoxic conditions

**Fig. 1:**
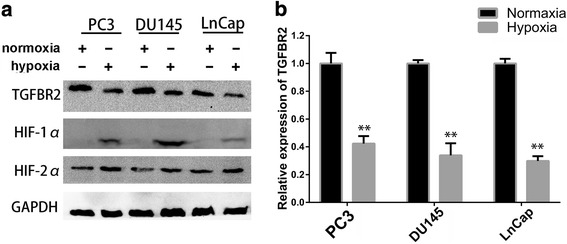
TGFBR2 expression was decreased under hypoxia. **a**. Prostate cancer cell lines (PC3, DU145, and LnCap) showed decreased levels of TGFBR2 protein in hypoxic condition, as measured by western blot. HIF-1α and HIF-2α expressions were elevated under hypoxia, while GAPDH was used as internal control. **b**. RT-PCR results also demonstrated that hypoxia treatment decreased the mRNA levels of TGFBR2 in PCa cell lines. **, *P* < 0.01

Recent studies have provided evidence that hypoxia promote cancer progression in prostate cancer, while the exact effects of hypoxia on EMT are not fully understood. As TGF-β signalling plays critical roles in PCa, we sought to study the impact of hypoxia on the pivotal receptor, TGFBR2. The Cancer Genome Atlas (TCGA) data showed that TGFBR2 expression was significantly reduced in prostate cancer tissues (Additional file 1: Figure S1). Since high level of hypoxia and decreased TGFBR2 expression has been reported to occur in human or rat PCa [15, 16], we hypothesized that hypoxia might at least partly contribute to low expression of TGFBR2 in PCa. In an attempt to test our hypothesis, we cultured three of prostate cancer cell lines, PC3, DU145 and LNCap, under hypoxic conditions to evaluate the impacts of hypoxia. 24 h after the hypoxic treatment, cells were harvested and the protein and mRNA expression levels of TGFBR2 were examined by western blot and RT-PCR, respectively. As shown in Fig. 1a and Additional file 1: Figure S4, compared to cells in normoxia, all cell lines in hypoxia showed decreased levels of TGFBR2 protein, while the well-established hypoxia indicator HIF-1α protein expression was elevated. RT-PCR results also demonstrated that hypoxia treatment decreased the mRNA levels of TGFBR2, which was in accordance with the western blot results (Fig. 1b). These results indicated that TGFBR2 expression was attenuated by hypoxia pathway. However, since the transcription factors (HIF-1α and HIF-2α) always activated the transcription of downstream gene with hypoxia response element (HRE, consensus sequence G/ACGTG) on their promoter region [20], which seemed that TGFBR2 was not likely to be a direct downstream molecule of hypoxia. As a result, we turned to find other hypoxia-regulated pathways which involved in TGFBR2 down-regulation.

### Hypoxia-induced EZH2 regulated TGFBR2 promoter hypermethylation and contributed to its epigenetic silencing in PCa

**Fig. 2:**
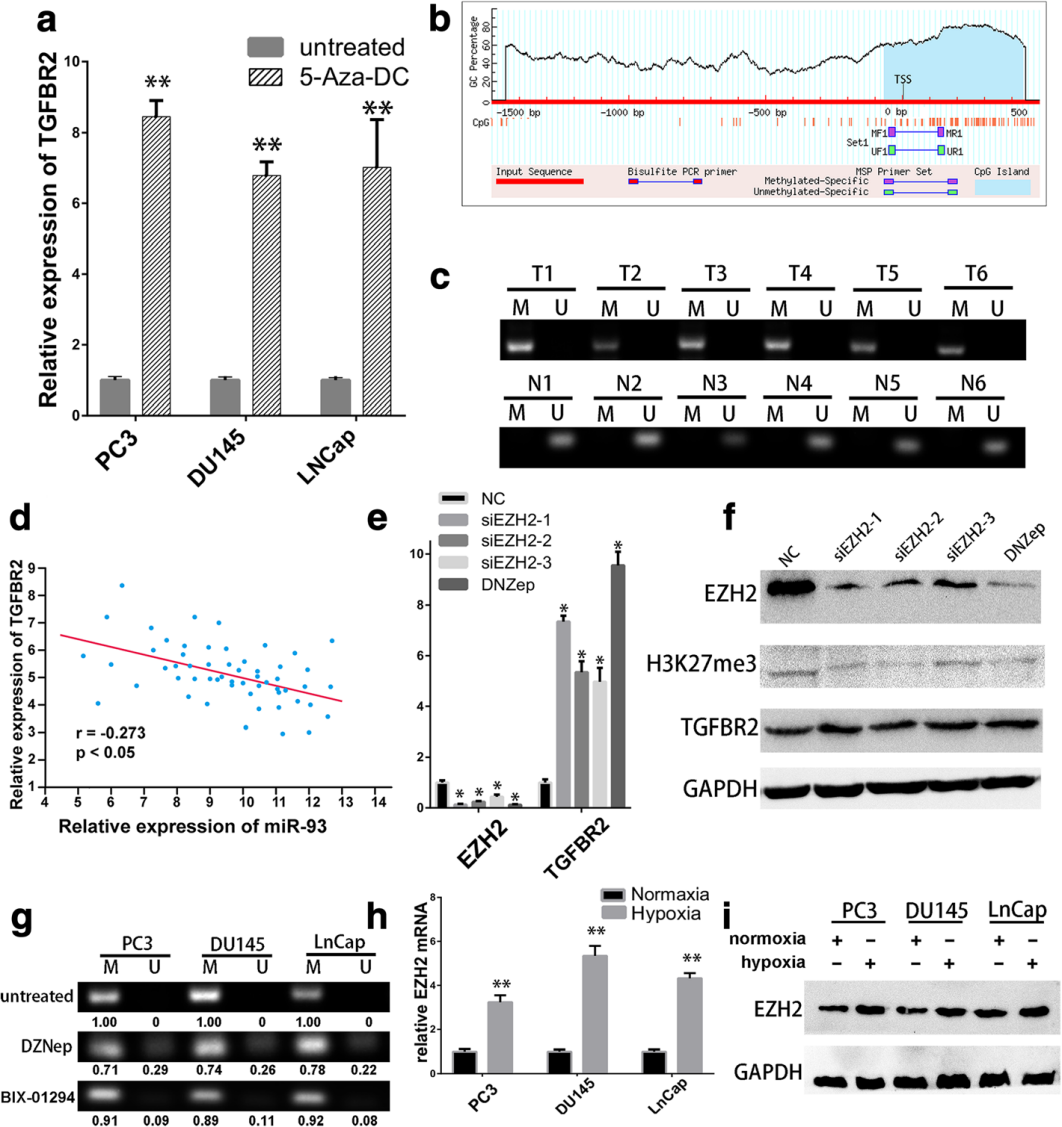
Hypoxia-induced EZH2 regulated TGFBR2 promoter hypermethylation and contributed to its epigenetic silencing in PCa. **a**. TGFBR2 expression in PCa cell lines elevated significantly after the 5-Aza-DC treatment via RT-qPCR. **b**. Graphic presentation of putative CpG island in promoter of TGFBR2 and primer for methylation specific PCR, as designed by MethPrimer website. **c**. Methylation specific PCR detected the methylation status of TGFBR2 promoter in six pairs of prostate cancer samples (T1-T6) and corresponding noncancerous tissues (N1-N6). M, methylation primer; U, unmethylation primer. **d**. Significant negative correlation between the expression of EZH2 and TGFBR2 via data from TCGA. **e-f**. The expression levels of TGFBR2 elevated significantly after treatment with siRNAs targeting EZH2 or EZH2 inhibitor DZNep, as detected by RT-qPCR (E) and western blot (F). **G**. Methylation specific PCR results showed DZNep and BIX-01294 partially decreased the methylation levels of PCa cell lines. **h-i**. Hypoxia induced both the mRNA (H) and protein expression (I) of EZH2.**, *P* < 0.01

One of the common reasons of transcriptional silencing of tumour suppressor genes in cancer is CpG-island promoter hypermethylation. Previous studies have shown that methylation silencing of TGFBR2 resulted in lower expression of TGFBR2 in rat prostate cancer. However, we further validated the epigenetic changes of TGFBR2 in human PCa. Firstly, we treated the three prostate cancer cell lines with 10 μmol/L of DNA demethylation agent 5-Aza-2′-deoxycytidine (5-Aza-DC), and detected the expression level of TGFBR2 by RT-PCR. As seen in Fig. 2a, the TGFBR2 expression in all cell lines elevated significantly after the 5-Aza-DC treatment, which implied the potential regulatory roles of DNA methylation. Besides, we detected the methylation status of TGFBR2 promoter in our six paired prostate tissues by methylation specific PCR (MSP). Using MethPrimer algorithm, we detected CpG-island around the presumed transcriptional start site (TSS) and designed the methylation (M) and unmethylation (U) primers (Fig. 2b). As shown in Fig. 2c, only methylated DNA molecules were detected in six prostate cancer tissues, while only unmethylated DNA molecules were identified in corresponding noncancerous tissues. As a result, methylation silencing of TGFBR2 in prostate cancers contributed at least partly the decreased expression of TGFBR2.

It reported that rat prostate cancer cell lines had increased H3K4me2 or H3K9me3 at the Tgfbr2 promoter, while human prostate cancer cell lines were likely to have increased H3K27me3 at the TGFBR2 promoter [15]. As a histone methyltransferase, the enhancer of zeste homolog 2 (EZH2) is proved to exert its primary function to methylate H3K27me by transferring methyl group. EZH2 has been proved to exert important oncogenic functions in PCa, TCGA data also implies that high expression of EZH2 was associated with worse overall survival and disease-free survival (Additional file 1: Figure S2). Therefore, we hypothesized that EZH2 induced the epigenetic silencing of TGFBR2 by H3K27me3 or other epigenetic modifications. Firstly, we examined the expression relationship of EZH2 and TGFBR2 using the Pearson correlation coefficient analysis. Our panel of 56 PCa tissues showed significant inverse correlation between the expression of EZH2 and TGFBR2 (*r* = − 0.273, *p* < 0.01) (Fig. 2d). By processing the data from TCGA, we also found significant negative correlation between the expression of these two molecules in 568 cases of PCa (*r* = − 0.3479, *p* < 0.0001) (Additional file 1: Figure S3). To further validate our hypothesis, we used siRNA targeting EZH2 or EZH2 inhibitor 3-deazaneplanocin (DZNep) to knockdown EZH2 expression in DU145 cell lines. After the reduction of EZH2 and H3K27 trimethylation, the mRNA and protein expression levels of TGFBR2 elevated significantly (Fig. 2e, f, Additional file 1: Figure S5). Moreover, we conducted MSP with prostate cancer cells which were treated with DZNep or not. The results in Fig. 2g illustrated that DZNep could partially decreased the methylation levels of cancer cell lines and helped the cells restore the hypomethylation status, as both methylated and unmethylated DNA molecules could be detected in DZNep-treated cells. Besides, other epigenetic modulators, such as H3k9 methylation inhibitor BIX-01294 [24], had only slight effects on MSP results, which also proved the importance of EZH2-mediated H3K27me3 on regulating the TGFBR2 promoter methylation. These combined results demonstrated that EZH2 regulated TGFBR2 promoter hypermethylation could contribute to its epigenetic silencing in PCa.

In an effort to explore the mechanism of hypoxia-induced TGFBR2 down-regulation, we further tried to evaluate the impact of hypoxia on EZH2. Previous study showed the expression of EZH2 could be regulated by HIF-1α/HIF-1β dependent hypoxic pathway in prostate cancer [25]. Therefore, we detected the EZH2 mRNA and protein expression of prostate cancer cells under normoxic or hypoxic conditions. As we could see in Fig. 2h, i and Additional file 1: Figure S6, hypoxia induced both the mRNA and protein expression of EZH2. Taken together, hypoxia-induced EZH2 mediated the epigenetic silencing of TGFBR2 in PCa through promoter hypermethylation.

### Hypoxia-responsive miR-93 was increased in PCa and correlated with cancer progression

**Fig. 3:**
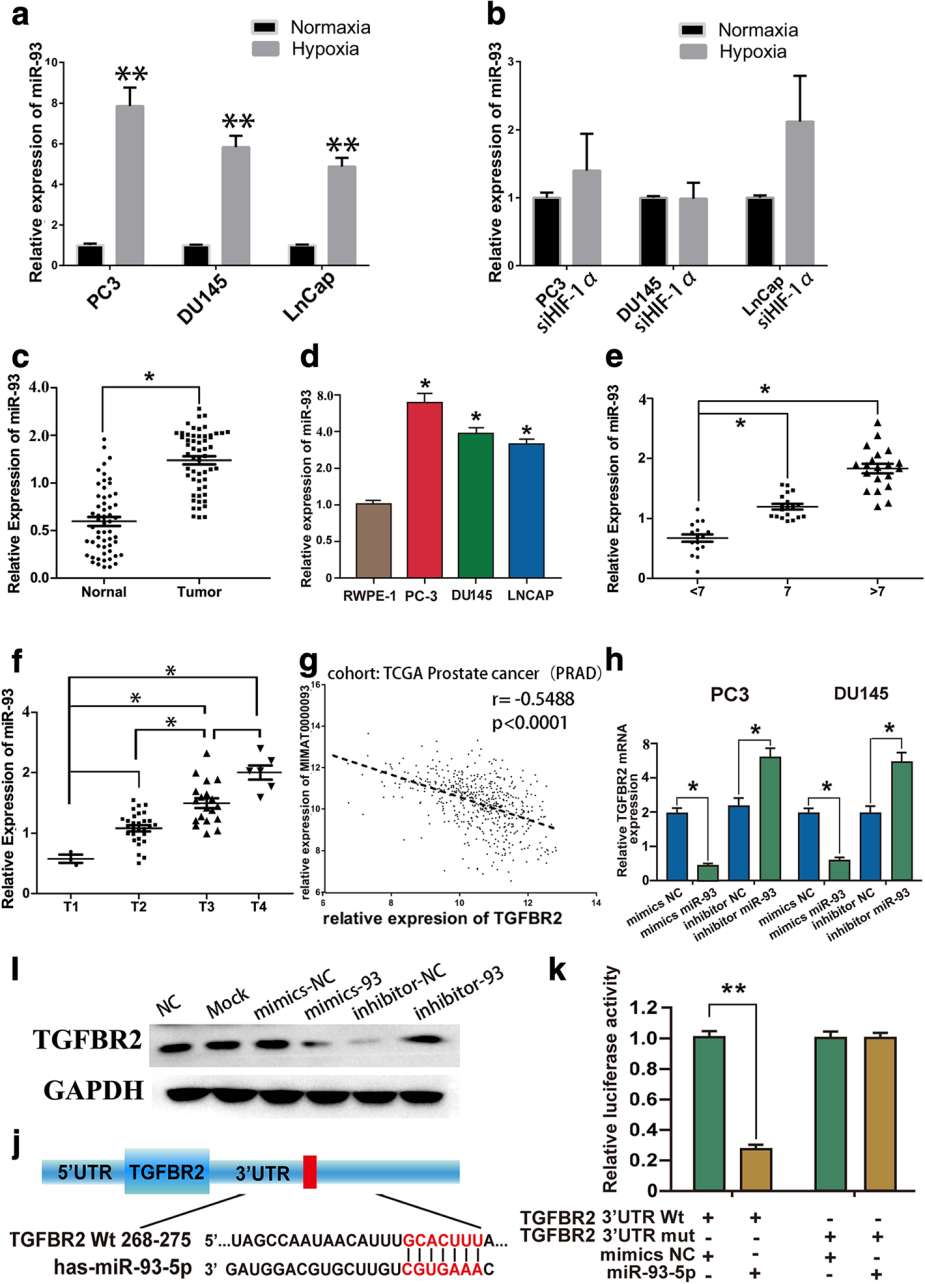
Hypoxia-induced miR-93 was upregulated in PCa with prognostic significance, while TGFBR2 was a bona fide direct target of miR-93. **a**. Compared to cells in normoxia, PCa cells in hypoxic conditions showed markedly increased expression of miR-93. **b**. Induction of miR-93 was attenuated under hypoxia in PCa cells after treatment of siRNA for HIF-1α. **c**. miR-93 expression levels are significantly elevated in PCa tissues in comparison to normal ones. **d**. Compared to normal prostate epithelial cell RWPE-1, PCa cell lines showed significant higher expression of miR-93. **e**. miR-93 expression levels were significantly increased in patients with Gleason score (GS) > 7 in comparison to the patients with GS ≤ 7. **f**. miR-93 expression levels were significantly increased in patients with higher tumor stage in comparison to the patients with lower one. **g**. Significant negative correlation between the expression of miR-93 (MIMAT0000093) and TGFBR2 in TCGA data. **h-i**. Both mRNA (H) and protein expression (I) of TGFBR2 were significantly decreased after transfection of miR-93 mimics, while TGFBR2 expression elevated after transfection of miR-93 inhibitor. **j**. Respective figure of seed sequence of TGFBR2 via TargetScan. **k**. Dual luciferase assay validated that miR-93 mimics silenced the wide-type TGFBR2 while had no inhibitory effect on seed sequence mutated TGFBR2. *, *P* < 0.01; **, *P* < 0.01

MicroRNAs (miRNAs) are a sort of small, endogenously expressed, well-conserved non-coding single stranded RNAs which can negatively regulate gene expression by binding directly to the 3′ untranslated region (3’-UTR) of corresponding target messenger RNAs (mRNAs) in a sequence-specific manner. Previous studies have identified a series of hypoxia-induced miRNAs in several cancers, such as miR-182 in PCa [22], and miR-25/93 in breast cancer [26]. Thus, we postulated that some deregulated hypoxia-responsive miRNAs might facilitate the PCa progression via reducing the expression of TGFBR2. By intersecting the potential miRNA-mRNA regulatory networks via an experimentally validated microRNA-target interactions database (miRTarBase) and well-known hypoxia-induced miRNAs in published articles, we extrapolated that miR-93 might be potential miRNAs which could regulate TGFBR2 expression and be induced by hypoxia. To validate whether miR-93 was a bona fide hypoxia-regulated miRNA in prostate cancer, we detected its expression in normoxic or hypoxic conditions. As shown in Fig. 3a, compared to cells in normoxia, expression of miR-93 was markedly increased in hypoxic conditions in all three PCa cell lines. To further confirm the results, we repeated the experiments in cells with siRNA-mediated HIF-1α knockdown, Additional file 1: Figure S7 showed that HIF-1α silencing was effective in prostate cancer cells, especially in DU145 and PC3. The experimental data showed that HIF-1α knockdown compromised the ability of hypoxia to induce miR-93 expression (Fig. 3b). These results combined together to confirm that miR-93 was a genuine target of hypoxia.

In an attempt to evaluate the potential of TGFBR2 as the direct target of miR-93, we firstly detected the expression pattern of miR-93 in PCa. We examined miR-93 expression levels in a panel of 45 human PCa tissues and found that the level of miR-93 was elevated in most of the malignant PCa tissues by 1.5 to 6 folds (Fig. 3c). Furthermore, compared to noncancerous prostate cells RWPE-1, the expression levels of miR-93 in three prostate cancer cell lines was significantly higher (Fig. 3d). To further investigate the clinical significance of elevated miR-93 expression, we assessed the association between miR-93 expression and several clinicopathologic characteristics of prostate cancer. Gleason score (GS) which reflects the differentiation and biological behavior of PCa is critical for PCa grading. In accordance with our prediction, higher GS accompanied with significantly higher expression of miR-93 (Fig. 3e). Besides, miR-93 expression levels are significantly elevated in patients of higher tumor stage (Fig. 3f). These findings demonstrated that higher expression of expression was correlated with the PCa progression and miR-93 had the potential to be useful biomarker for PCa.

### TGFBR2 was a direct target of miR-93 in prostate cancer

To figure out if hypoxia-responsive miR-93 contributed to the attenuation of TGFBR2 expression via targeting the 3’-UTR of TGFBR2 mRNA, we performed correlation coefficient analysis between miR-93 and TGFBR2 using data of TCGA. We found significant negative correlation between the expression of miR-93 (MIMAT0000093) and TGFBR2 in 568 cases of PCa (*r* = − 0.5488, *p* < 0.0001) (Fig. 3g). Then we transfected the synthetical mimics and inhibitor of miR-93 into two PCa cell lines. As shown in Fig. 3h, i and Additional file 1: Figure S8, after transfection of miR-93 mimics, both mRNA and protein expression of TGFBR2 were significantly decreased, while TGFBR2 expression elevated after transfection of miR-93 inhibitor. According to seed sequence data of TGFBR2 via TargetScan (Fig. 3j), we constructed dual luciferase reporter of wild-type or mutant 3’-UTR of TGFBR2 mRNA and performed a dual luciferase reporter assay. As seen in Fig. 3k, miR-93 mimics had the ability to decrease the relative luciferase activity of wild-type TGFBR2, while the inhibitory effects were abolished when the seed sequence of TGFBR2 3’-UTR was mutated. These results proved that TGFBR2 was exactly the target of miR-93 in prostate cancer cells.

### MiR-93 functions as an oncogenic miRNA in prostate cancer

**Fig. 4:**
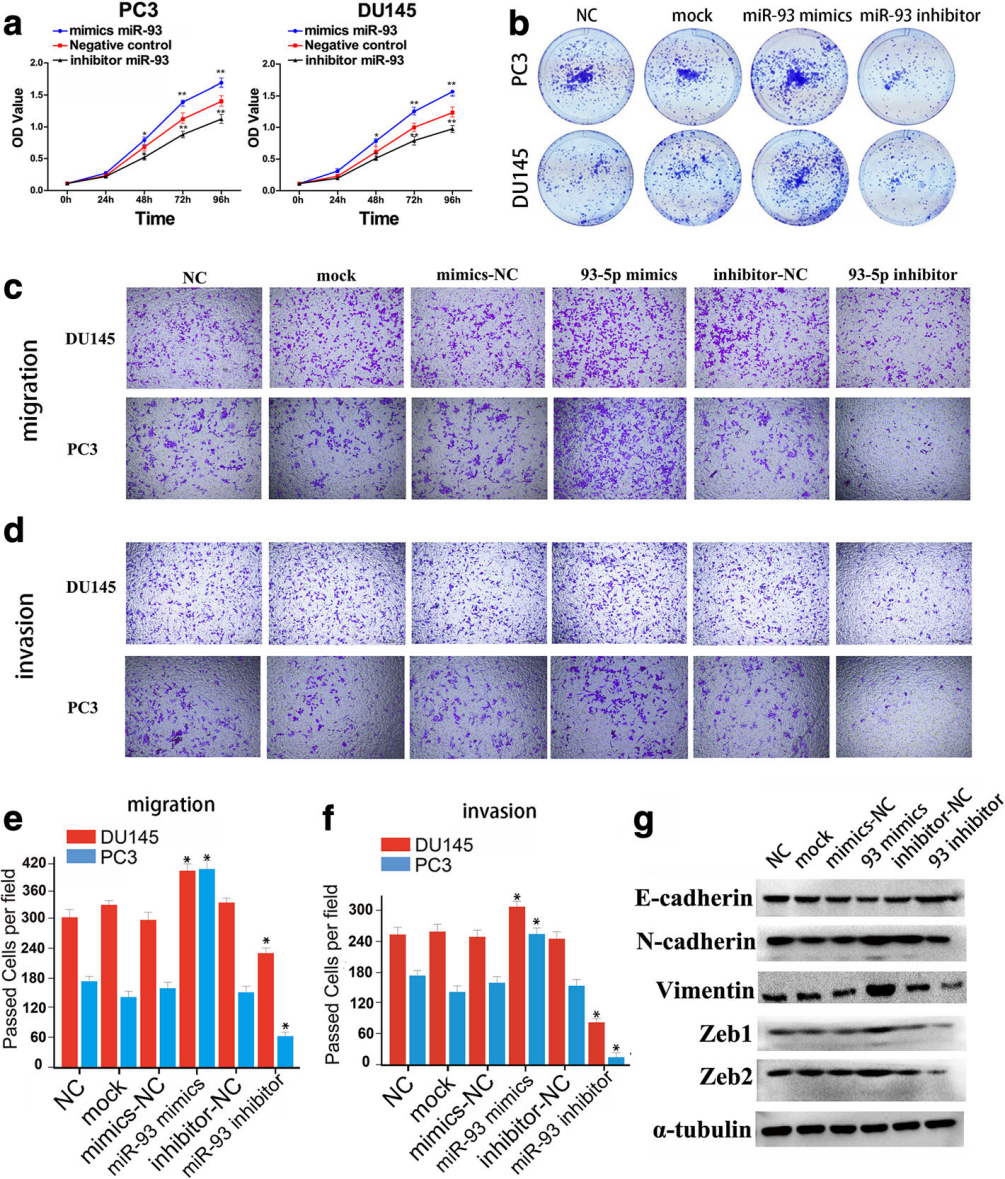
miR-93 exerted oncogenic functions in PCa. **a**. MTS assay showed that the proliferation rate of PC3 and DU145 cells was significantly repressed after silencing of miR-93, while the cell proliferation was dramatically accelerated following overexpression of miR-93. **b**. Representative photographs of the colony formation assay showed miR-93 mimics promoted PCa cell survival and proliferation. **c-d**. Transwell assay showed high expression of miR-93 promoted PCa cell migration (C) and invasion (D). **e-f**. Column figures using data from Transwell assay showed high expression of miR-93 promoted PCa cell migration (E) and invasion (F) in two PCa cell lines. **g**. Western blot results showed the regulatory functions of miR-93 expression on epithelial-mesenchymal markers in DU145 cells. *, *P* < 0.01; **, *P* < 0.01

To explore the role of miR-93 in prostate cancer cells, we transfected PC3 and DU145 with miR-93 mimics or inhibitor to upregulate or decrease miR-93 expression, respectively. MTS assay showed that the proliferation rate of PC3 and DU145 cells was significantly repressed after silencing of miR-93, while the cell proliferation was dramatically accelerated following overexpression of miR-93 (Fig. 4a). Furthermore, the ability of colony formation was also affected by miR-93 mimics or antagonist in same manner of that of MTS (Fig. 4b, Additional file 1: Figure S9).

To determine whether miR-93could regulate the epithelial-mesenchymal transition of PCa cells, we performed in vitro gain-of-function or loss-of-function analyses by overexpressing or silencing miR-93 in PC3 and DU145 cells, and detected the effects of miR-93 on cell migration and invasion. Transwell migration and invasion assays were performed, which showed that ectopic expression of miR-93 significantly promoted the migration and invasion of PC3 and DU145 cells (Fig. 4c-f). In contrast, the migration and invasion ability of PCa cells was suppressed when endogenous miR-93 was silenced with miR-93 specific inhibitors (Fig. 4c-f). Besides, western blot and RT-qPCR showed that miR-93 mimics decreased the expression of epithelial marker E-cadherin, while increasing the expression of mesenchymal markers such as N-cadherin, Vimentin, Zeb1/2. In addition, miR-93 inhibitor exerted inversed regulatory roles on EMT markers (Fig. 4g, Additional file 1: Figure S10). These observations suggested that miR-93 could promote PCa cell migration and invasion in vitro.

### TGFBR2 mediated the oncogenic functions of miR-93 in functional rescue assays

**Fig. 5:**
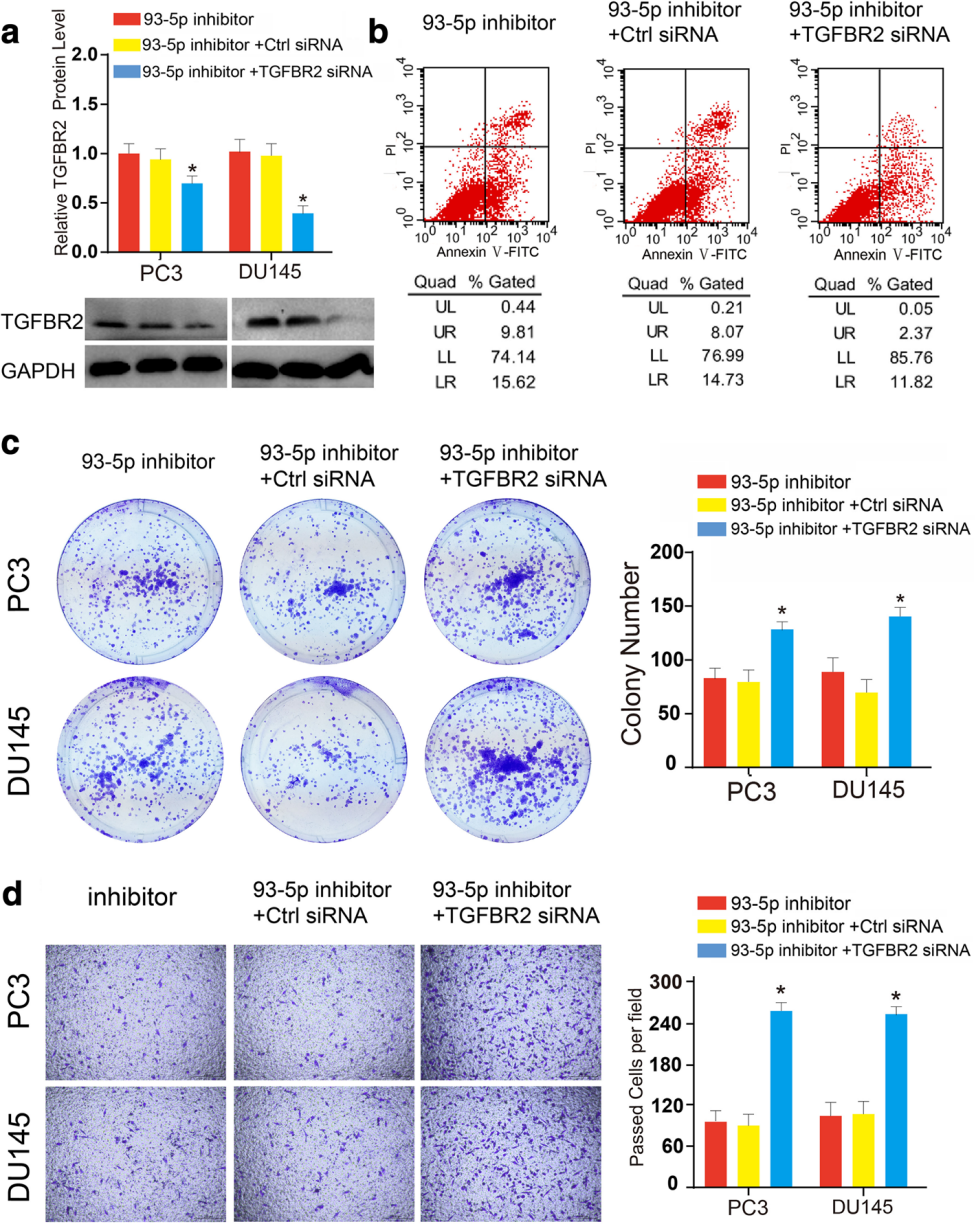
Rescue assays validated that TGFBR2 mediated the oncogenic functions of miR-93. **a**. The expression of TGFBR2 was shown after treatment of miR-93 mimics with or without siRNA for TGFBR2, as detected by RT-qPCR and Western blot. **b**. Annexin V-FITC apoptotic assays for treated cells. Additional TGFBR2 silencing decreased the cell ratio of early apoptosis in PC3 cells. **c**. Colony formation assay showed TGFBR2 siRNA resulted in increased capability of colony formation in two PCa cell lines with miR-93 inhibitor. **d**. Transwell assay showed TGFBR2 siRNA resulted in increased capability of cell migration in two PCa cell lines with miR-93 inhibitor. *, *P* < 0.01; **, *P* < 0.01

As we had shown that miR-93 silencing could decrease the proliferation, migration, and invasion of PCa cells, we were curious that if TGFBR2 could mediate the oncogenic functions of miR-93 and if TGFBR2 silencing could restore these functions of PCa cells. Thus, we conducted a series of functional rescue assays to settle these puzzles. Cells which were co-transfected with miR-93 inhibitor and TGFBR2 siRNA showed significantly lower mRNA and protein expression of TGFBR2, compared to cells with single transfection of miR-93 inhibitor or additional negative control (Fig. 5a). These cells were then analyzed by flow cytometry apoptosis assay, which showed that additional TGFBR2 silencing decreased the cell ratio of early apoptosis in PC3 cells (Fig. 5b). Besides, TGFBR2 interference resulted in increased capability of colony formation in two PCa cell lines (Fig. 5c), which was paralleled with the effects of miR-93 overexpression in PCa cell lines. Subsequently, to test whether abrogation of TGFBR2 could rescue the pro-tumorigenic effects of miR-93, we conducted the Transwell migration essays. We found that TGFBR2 blocking promoted the restoration of migratory ability of PCa which were inhibited by miR-93 inhibitor (Fig. 5d). Taken together, our data presented here supported our hypothesis that abrogation of TGFBR2 restored the oncogenic functions of PCa cells which were compromised by miR-93 silencing, and cellular functions of miR-93 were mediated at least partly by directly targeting TGFBR2.

## Discussion

**Fig. 6:**
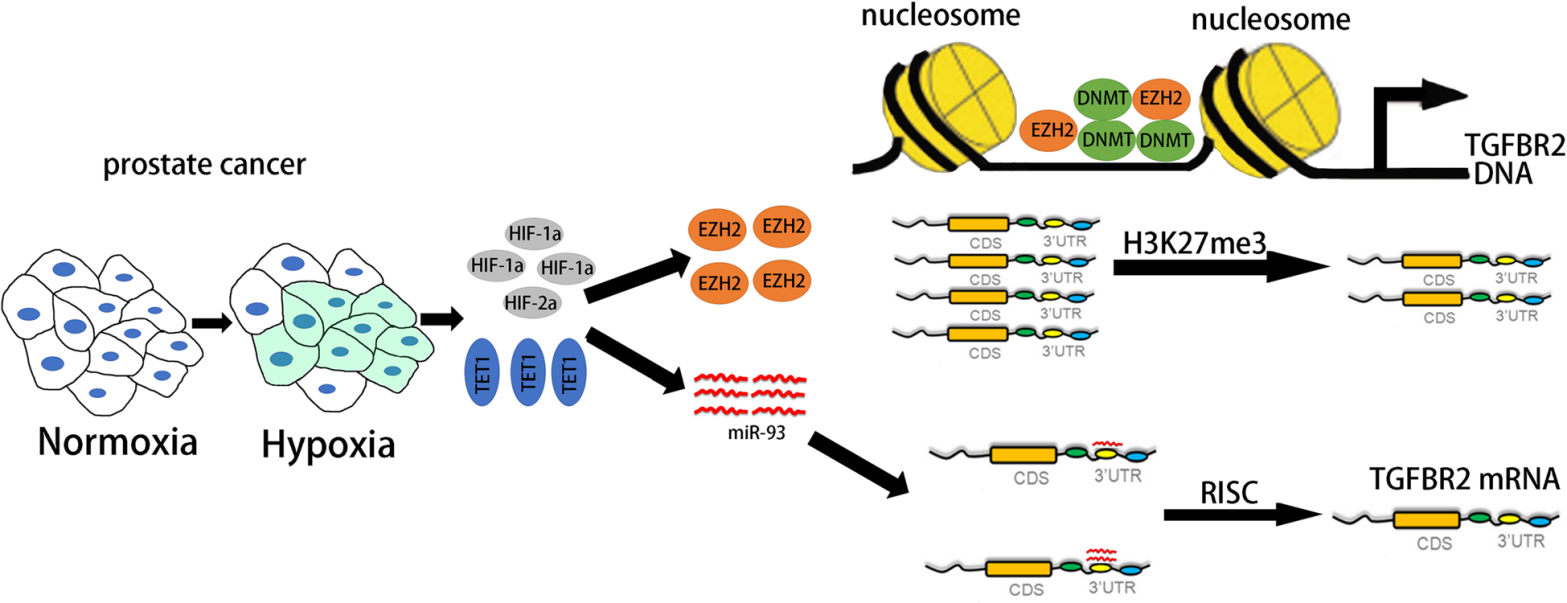
Proposed working model of hypoxia-EZH2/miR-93-TGFBR2 axis in prostate cancer progression. Prostate cancer generates a hypoxic microenvironment which induces the expression of a series of downstream genes such as HIF-1α, HIF-1β, and TET1, which further promotes the upregulation of epigenetic modulator EZH2 and hypoxia-responsive miR-93. EZH2 may catalyze H3K27me3 or recruit several DNA methyltransferases, which results in higher level of promoter methylation of TGFBR2 gene and subsequent epigenetic silencing of TGFBR2. Besides, miR-93 may reduce the expression of TGFBR2 via binding to 3’-UTR of TGFBR2 mRNA, which can further repress the expression of TGFBR2

Identifying the mechanisms underlying carcinogenesis and tumor progression remains a great challenge for the current study of prostate cancer. The present study demonstrated that hypoxia could attenuated the expression of TGFBR2 to promote prostate cancer progression via diverse cellular pathways. We propose that prostate cancer generates a hypoxic microenvironment, and hypoxia induces the expression of a series of downstream genes such as hypoxia-inducible factors and ten-eleven translocation 1 (TET1), which promotes the upregulation of epigenetic modulator EZH2 and hypoxia-responsive miR-93. The histone-lysine N-methyltransferase enzyme EZH2 can catalyze the trimethylation of H3K27 (H3K27me3) or recruit several DNA methyltransferases, which results in higher level of promoter methylation of TGFBR2 gene and subsequent epigenetic silencing of TGFBR2. Besides, hypoxia-regulated miR-93 can reduce the expression of TGFBR2 via binding to 3’-UTR of TGFBR2 mRNA and form the RNA-induced silencing complex (RISC), which can further repress the expression of TGFBR2. A proposed working model of hypoxia-EZH2/miR-93-TGFBR2 axis in prostate cancer progression is presented in Fig. 6.

The first important finding of our study is that hypoxia reduces the expression of TGFBR2 in prostate cancer. Tumor microenvironmental changes such as hypo-nutrition and hypoxia can force cancer cells to develop strategies which can help them adapt to adverse conditions or provide growth advantage for them via regulating specific cell signaling. For example, EpCAM-CD98hc-CD147 complex can regulate the Akt/mTOR/AMPK signaling through stablizing the amino acid transporter LAT1 and changing the susceptibility of PCa stem-like cells to EGF, and finally promote the adaption to hypo-nutrient condition [27, 28]. TGF-β signaling is pivotal for tissue development in normal cells and aberrant proliferation and EMT in cancer cells [29, 30], as a critical modulator of TGF-β signaling, the deregulation of TGFBR2 has the potential to affect a variety of biological processes of prostate cancer cells. Previous transgenic mice with conditional stromal knockout of TGFBR2 were inclined to promote tumorigenesis as a result of Wnt3a upregulation, which highlighted the suppressive roles of TGFBR2 in TGF-β/Wnt signaling axis [13]. Furthermore, TGF-β/Wnt signaling following the knockout of TGFBR2 enabled the prostatic intraepithelial neoplasia lesions to progress to adenocarcinoma and even facilitated the epithelia to become resistant to androgen ablation [31]. Cancer cells undergo complex biological responses when placed in hypoxia microenvironment, including activation of hypoxia-responsible genes and changes of signaling pathways that affect cell proliferation, EMT, and angiogenesis. Cancer cells have the ability to adjust to and exploit these downstream molecules and signaling pathways, promoting their growth and evading the restriction of immune system [32]. As for prostate cancer cells, previous findings have validated the existence of hypoxic conditions in cancer tissues and several biomarkers of tumor hypoxia and angiogenesis are even capable of predicting the clinical outcomes of radiotherapy for localized PCa [18]. Therefore, identifying the regulatory relationship of hypoxia and TGFBR2 is of paramount importance for understanding the crosstalk between TGF-β/Wnt signaling and hypoxia-response pathway in PCa.

Among multiple hypoxia-response pathway, the HIF-dependent pathway is the most prominent one. HIFs have the ability to bind to hypoxia responsive elements (HREs) sites of target genes, which activates the transcription of their targets [33]. However, we did not identify any HREs around the promoter of TGFBR2 gene, which implied more underlying mechanisms might be involved. Epigenetic regulation has become an emerging field of gene expression regulation, and higher level of DNA methylation especially in CpG island of gene promoter has resulted in gene silencing in cancers [34].

Our experiments showed that hypermethylation in TGFBR2 promoter reduced the expression of TGFBR2, which was in accordance with previous findings that Tgfbr2 underwent methylation silencing in rat prostate cancers [15, 35]. Epigenetic markers provide us important clues to identify critical modulators, the relatively common epigenetic marker H3K27me3 in human prostate cancer cell lines imply the involvement of H3K27 enzyme EZH2 [15]. Significant negative correlation between EZH2 and TGFBR2 also consolidated our hypothesis that EZH2 regulated the expression of TGFBR2. Our results validated the induction of EZH2 expression upon hypoxia treatment, and provided evidences that silencing of EZH2 could partially rescue the expression of TGFBR2. These results, together with findings which proved the upregulation of EZH2 in cultured endothelial cells exposed to hypoxia [36], formed a hypoxia-EZH2-TGFBR2 axis in regulating the expression of TGFBR2.

MiRNAs are well-conserved non-coding single stranded RNAs which play vital roles in many biological processes of tumors. As master posttranscriptional regulators, miRNAs can negatively regulate gene expression by binding directly to the 3’-UTR of corresponding target mRNAs in a sequence-specific manner, forming RISC and inducing mRNA degradation or protein translation repression [37]. Recent evidence reveals that a variety of miRNAs can functions as tumor enhancers or suppressors in the evolution of tumor progression in PCa [38]. Besides, hypoxic tumor microenvironment contributes to important hallmarks of cancer phenotypes through regulating a panel of downstream molecules including miRNAs, while miRNAs can also regulate the expression and stabilization of hypoxia-inducible factors. The interaction between miRNAs and HIFs accounts for many critical events during tumorigenesis, therefore, the efforts to clarify the machinery how miRNAs interact with hypoxia in tumor cells are of paramount importance [37, 39]. MiR-93 has been frequently reported to have aberrant expression in various malignant tumors, it’s also been identified as a member of a pan-cancer, co-regulated oncogenic miRNA ‘superfamily’ which co-targets important tumor suppressive genes by a central GUGC core motif [40]. Besides, miR-93 expression was found to markedly increase in breast cancer under hypoxia conditions in a HIF-1α-independent manner, mediating the hypoxic regulation with direct TET1 binding [26]. Nevertheless, the definite functions and regulatory mechanisms of miR-93 in PCa still remains largely unknown. Our experimental data showed that TGFBR2 was a bona fide target of hypoxia-regulated miR-93 which exerted oncogenic roles in the carcinogenesis and development of PCa. A series of in vitro assays and rescue experiments proved that TGFBR2 regulation mediated the oncogenic functions of miR-93 in PCa. Furthermore, we identified the significant correlations between miR-93 and several clinicopathologic characteristics and clinical prognosis of PCa, which highlighted the potential of miR-93 as a promising biomarker in evaluating the aggressiveness of PCa and predictive survival of PCa patients.

## Conclusions

In conclusion, hypoxic condition in prostate cancer induces the expression of EZH2 and miR-93, which initiates H3K27me3 in TGFBR2 promoter and microRNA-induced silencing of target gene respectively. These diverse pathways regulated by hypoxia eventually contribute to attenuation of TGFBR2 expression and promote tumor progression in PCa.

## Additional file

Additional file 1: **Figure S1.** TGFBR2 expression was significantly reduced in prostate cancer tissues from data of TCGA. **Figure S2.** Kaplan-Meier overall survival and disease-free survival analysis for EZH2 using PRAD TCGA dataset. **Figure S3.** EZH2 and TGFBR2 expression were inversely correlated in PCa patients using PRAD TCGA dataset. **Figure S4.** Bar diagrams which represented the relative protein expression levels of TGFBR2 (A), HIF1A (B), and HIF2A (C) in normoxic or hypoxic condition. **Figure S5.** Bar diagrams which represented the relative protein expression levels of EZH2 (A), H3k27me3 (B), and TGFBR2 (C) after treating with EZH2 siRNAs or DNZep. **Figure S6.** Bar diagrams which represented the relative protein expression levels of EZH2 in normoxia or hypoxia. **Figure S7.** Western blot showed that siRNA decreased the protein expression level of HIF-1a, especially in DU145 and PC3 cells. **Figure S8.** Bar diagrams which represented the relative protein expression levels of TGFBR2 after miR-93 overexpression or downregulation. **Figure S9.** Bar diagrams which represented the colony numbers in different groups after miR-93 overexpression or downregulation. **Figure S10.** Bar diagrams which represented the relative mRNA expression levels of E-cadherin (A), N-cadherin (B), Vimentin (C), Zeb1 (D), and Zeb2 (E) after treating with miR-93 mimics or inhibitor. **Table S1.** Clinicopathological characters in our cohort of 56 PCa patients. **Table S2.** Primer list used in this study. (DOC 1410 kb)

## Abbreviations

3′-UTR: 3′-untranslated regions
5-Aza-DC: 5-aza-2′-deoxycytidine
AR: Androgen receptor
CRPC: Castration-resistant prostate cancer
DZNep: 3-deazaneplanocin
EMT: Epithelial-mesenchymal transition
EZH2: Enhancer of zeste homolog 2
GS: Gleason score
HIF-1α: Hypoxia-inducible factor 1α
MSP: Methylation specific polymerase chain reaction
PCa: Prostate cancer
RISC: RNA-induced silencing complex
RT-qPCR: Real-time quantitative polymerase chain reaction
TCGA: The Cancer Genome Atlas
TET1: Ten-eleven translocation 1
TGFBR2: Type II TGF-β receptor
TGF-β: Transforming growth factor β

## References

[1] Zhang K, Bangma CH, Roobol MJ (2017). Prostate cancer screening in Europe and Asia. Asian J Urol.

[2] Siegel RL, Miller KD, Jemal A (2017). Cancer statistics, 2017. CA Cancer J Clin.

[3] Litwin MS, Tan HJ (2017). The diagnosis and treatment of prostate cancer: a review. JAMA.

[4] Gillessen S, Omlin A, Attard G, de Bono JS, Efstathiou E, Fizazi K, Halabi S, Nelson PS, Sartor O, Smith MR (2015). Management of patients with advanced prostate cancer: recommendations of the St Gallen advanced prostate cancer consensus conference (APCCC) 2015. Ann Oncol.

[5] Gartrell BA, Coleman R, Efstathiou E, Fizazi K, Logothetis CJ, Smith MR, Sonpavde G, Sartor O, Saad F (2015). Metastatic prostate cancer and the bone: significance and therapeutic options. Eur Urol.

[6] Logothetis CJ, Gallick GE, Maity SN, Kim J, Aparicio A, Efstathiou E, Lin SH (2013). Molecular classification of prostate cancer progression: foundation for marker-driven treatment of prostate cancer. Cancer Discov.

[7] Bostrom PJ, Bjartell AS, Catto JW, Eggener SE, Lilja H, Loeb S, Schalken J, Schlomm T, Cooperberg MR (2015). Genomic predictors of outcome in prostate cancer. Eur Urol.

[8] Savagner P. Epithelial-mesenchymal transitions: from cell plasticity to concept elasticity. Curr Top Dev Biol. 2015;112:273–300. 10.1016/bs.ctdb.2014.11.021 25733143

[9] Nakazawa M, Kyprianou N. Epithelial-mesenchymal-transition regulators in prostate cancer: androgens and beyond. J Steroid Biochem Mol Biol. 2017;166:84–90. 10.1016/j.jsbmb.2016.05.007 27189666

[10] Reddi AH, Roodman D, Freeman C, Mohla S (2003). Mechanisms of tumor metastasis to the bone: challenges and opportunities. J Bone Miner Res.

[11] Meng X, Vander Ark A, Lee P, Hostetter G, Bhowmick NA, Matrisian LM, Williams BO, Miranti CK, Li X (2016). Myeloid-specific TGF-beta signaling in bone promotes basic-FGF and breast cancer bone metastasis. Oncogene.

[12] Zhang W, Zhang T, Jin R, Zhao H, Hu J, Feng B, Zang L, Zheng M, Wang M. MicroRNA-301a promotes migration and invasion by targeting TGFBR2 in human colorectal cancer. J Exp Clin Cancer Res. 2014;33:113. 10.1186/s13046-014-0113-6 PMC4304202 25551793

[13] Li X, Placencio V, Iturregui JM, Uwamariya C, Sharif-Afshar AR, Koyama T, Hayward SW, Bhowmick NA (2008). Prostate tumor progression is mediated by a paracrine TGF-beta/Wnt3a signaling axis. Oncogene.

[14] Bjerke GA, Pietrzak K, Melhuish TA, Frierson HF, Paschal BM, Wotton D (2014). Prostate cancer induced by loss of Apc is restrained by TGFbeta signaling. PLoS One.

[15] Yamashita S, Takahashi S, McDonell N, Watanabe N, Niwa T, Hosoya K, Tsujino Y, Shirai T, Ushijima T (2008). Methylation silencing of transforming growth factor-beta receptor type II in rat prostate cancers. Cancer Res.

[16] Mishra S, Deng JJ, Gowda PS, Rao MK, Lin CL, Chen CL, Huang T, Sun LZ (2014). Androgen receptor and microRNA-21 axis downregulates transforming growth factor beta receptor II (TGFBR2) expression in prostate cancer. Oncogene.

[17] Ayub SG, Kaul D, Ayub T. An androgen-regulated miR-2909 modulates TGFbeta signalling through AR/miR-2909 axis in prostate cancer. Gene. 2017;631:1–9. 10.1016/j.gene.2017.07.037 28754634

[18] Vergis R, Corbishley CM, Norman AR, Bartlett J, Jhavar S, Borre M, Heeboll S, Horwich A, Huddart R, Khoo V (2008). Intrinsic markers of tumour hypoxia and angiogenesis in localised prostate cancer and outcome of radical treatment: a retrospective analysis of two randomised radiotherapy trials and one surgical cohort study. Lancet Oncol.

[19] Ranasinghe WK, Sengupta S, Williams S, Chang M, Shulkes A, Bolton DM, Baldwin G, Patel O (2014). The effects of nonspecific HIF1alpha inhibitors on development of castrate resistance and metastases in prostate cancer. Cancer Med.

[20] Tong D, Liu Q, Liu G, Yuan W, Wang L, Guo Y, Lan W, Zhang D, Dong S, Wang Y (2016). The HIF/PHF8/AR axis promotes prostate cancer progression. Oncogene.

[21] Marhold M, Tomasich E, El-Gazzar A, Heller G, Spittler A, Horvat R, Krainer M, Horak P (2015). HIF1alpha regulates mTOR signaling and viability of prostate cancer stem cells. Mol Cancer Res.

[22] Li Y, Zhang D, Wang X, Yao X, Ye C, Zhang S, Wang H, Chang C, Xia H, Wang YC, et al. Hypoxia-inducible miR-182 enhances HIF1alpha signaling via targeting PHD2 and FIH1 in prostate cancer. Sci Rep. 2015;5:12495. 10.1038/srep12495 PMC4513346 26205124

[23] Cao P, Deng Z, Wan M, Huang W, Cramer SD, Xu J, Lei M, Sui G. MicroRNA-101 negatively regulates Ezh2 and its expression is modulated by androgen receptor and HIF-1alpha/HIF-1beta. Mol Cancer. 2010;9:108. 10.1186/1476-4598-9-108 PMC2881117 20478051

[24] Kubicek S, O'Sullivan RJ, August EM (2007). Reversal of H3K9me2 by a small-molecule inhibitor for the G9a histone methyltransferase. Mol Cell.

[25] Varambally S, Cao Q, Mani RS, Shankar S, Wang X, Ateeq B, Laxman B, Cao X, Jing X, Ramnarayanan K (2008). Genomic loss of microRNA-101 leads to overexpression of histone methyltransferase EZH2 in cancer. Science.

[26] Wu MZ, Cheng WC, Chen SF, Nieh S, O'Connor C, Liu CL, Tsai WW, Wu CJ, Martin L, Lin YS (2017). miR-25/93 mediates hypoxia-induced immunosuppression by repressing cGAS. Nat Cell Biol.

[27] Yoshida GJ, Saya H (2014). EpCAM expression in the prostate cancer makes the difference in the response to growth factors. Biochem Biophys Res Commun.

[28] Xu D, Hemler ME (2005). Metabolic activation-related CD147-CD98 complex. Mol Cell Proteomics.

[29] de Miranda NF, van Dinther M, van den Akker BE, van Wezel T, ten Dijke P, Morreau H (2015). Transforming growth factor beta signaling in colorectal cancer cells with microsatellite instability despite Biallelic mutations in TGFBR2. Gastroenterology.

[30] Oshima H, Nakayama M, Han TS, Naoi K, Ju X, Maeda Y, Robine S, Tsuchiya K, Sato T, Sato H (2015). Suppressing TGFbeta signaling in regenerating epithelia in an inflammatory microenvironment is sufficient to cause invasive intestinal cancer. Cancer Res.

[31] Placencio VR, Sharif-Afshar AR, Li X, Huang H, Uwamariya C, Neilson EG, Shen MM, Matusik RJ, Hayward SW, Bhowmick NA (2008). Stromal transforming growth factor-beta signaling mediates prostatic response to androgen ablation by paracrine Wnt activity. Cancer Res.

[32] Harris AL (2002). Hypoxia--a key regulatory factor in tumour growth. Nat Rev Cancer.

[33] Semenza GL (2012). Hypoxia-inducible factors: mediators of cancer progression and targets for cancer therapy. Trends Pharmacol Sci.

[34] Esteller M (2007). Cancer epigenomics: DNA methylomes and histone-modification maps. Nat Rev Genet.

[35] Banerjee J, Mishra R, Li X, Jackson RS, Sharma A, Bhowmick NA (2014). A reciprocal role of prostate cancer on stromal DNA damage. Oncogene.

[36] Mitic T, Caporali A, Floris I, Meloni M, Marchetti M, Urrutia R, Angelini GD, Emanueli C (2015). EZH2 modulates angiogenesis in vitro and in a mouse model of limb ischemia. Mol Ther.

[37] Rupaimoole R, Calin GA, Lopez-Berestein G, Sood AK (2016). miRNA deregulation in cancer cells and the tumor microenvironment. Cancer Discov.

[38] Fabris L, Ceder Y, Chinnaiyan AM, Jenster GW, Sorensen KD, Tomlins S, Visakorpi T, Calin GA (2016). The potential of MicroRNAs as prostate cancer biomarkers. Eur Urol.

[39] Shen G, Li X, Jia YF, Piazza GA, Xi Y (2013). Hypoxia-regulated microRNAs in human cancer. Acta Pharmacol Sin.

[40] Hamilton MP, Rajapakshe K, Hartig SM, Reva B, McLellan MD, Kandoth C, Ding L, Zack TI, Gunaratne PH, Wheeler DA, et al. Identification of a pan-cancer oncogenic microRNA superfamily anchored by a central core seed motif. Nat Commun. 2013;4:2730. 10.1038/ncomms3730 PMC3868236 24220575

