# Supplementary material for: Attenuation of TGFBR2 expression and tumour progression in prostate cancer involve diverse hypoxia-regulated pathways
Source: J Exp Clin Cancer Res. 2018 Apr 27;37:89. doi: 10.1186/s13046-018-0764-9 (PMC5921809; doi:10.1186/s13046-018-0764-9)
Supplement: Supplementary file 1 — Figure S1. TGFBR2 expression was significantly reduced in prostate cancer tissues from data of TCGA. Figure S2. Kaplan-Meier overall survival and disease-free survival analysis for EZH2 using PRAD TCGA dataset. Figure S3. EZH2 and TGFBR2 expression were inversely correlated in PCa patients using PRAD TCGA dataset. Figure S4. Bar diagrams which represented the relative protein expression levels of TGFBR2 (A), HIF1A (B), and HIF2A (C) in normoxic or hypoxic condition. Figure S5. Bar diagrams which represented the relative protein expression levels of EZH2 (A), H3k27me3 (B), and TGFBR2 (C) after treating with EZH2 siRNAs or DNZep. Figure S6. Bar diagrams which represented the relative protein expression levels of EZH2 in normoxia or hypoxia. Figure S7. Western blot showed that siRNA decreased the protein expression level of HIF-1a, especially in DU145 and PC3 cells. Figure S8. Bar diagrams which represented the relative protein expression levels of TGFBR2 after miR-93 overexpression or downregulation. Figure S9. Bar diagrams which represented the colony numbers in different groups after miR-93 overexpression or downregulation. Figure S10. Bar diagrams which represented the relative mRNA expression levels of E-cadherin (A), N-cadherin (B), Vimentin (C), Zeb1 (D), and Zeb2 (E) after treating with miR-93 mimics or inhibitor. Table S1. Clinicopathological characters in our cohort of 56 PCa patients. Table S2. Primer list used in this study. (DOC 1410 kb) [file 13046_2018_764_MOESM1_ESM.doc]

**Supplementary**

**Attenuation of TGFBR2 Expression and Tumour Progression in Prostate Cancer Involve Diverse Hypoxia-Regulated Pathways**

**Authors:**

**Hui Zhou^1,2+^, Guanqing Wu^1,2,3+^, Xueyou Ma^1,2+^, Jun Xiao^1,2^, Gan Yu^1,2^, Chunguang** **Yang^1,2^, Nan Xu^1,2^, Bao Zhang^3^, Jun Zhou^4^, Zhangqun Ye^1,2^, Zhihua Wang^1,2*^**

1. Department of Urology, Tongji Hospital, Tongji Medical College, Huazhong University of Science and Technology, Wuhan 430030, China

2. Institute of Urology, Tongji Hospital, Tongji Medical College, Huazhong University of Science and Technology, Wuhan, China.

3. Department of Urology, Beijing Aerospace General Hospital, Beijing, 100076, China.

4. Department of Urology, The third people Hospital of Hubei Province, Wuhan, 430030, China

+ these authors contributed equally to this work.

^*^ Correspondence to Professor Zhihua Wang, Email: zhwang_hust@hotmail.com.

**Correspondence:** Professor Zhihua Wang, Department of Urology, Tongji Hospital, Tongji Medical College, Huazhong University of Science and Technology, Wuhan 430030, China. Institute of Urology of Hubei Province, Wuhan 430030, China. Email: zhwang_hust@hotmail.com; Phone: 86-27-8366-5308; Fax: 86-27-8366-5368.

**Supplementary Legends**

**Fig. S1. TGFBR2 expression was significantly reduced in prostate cancer tissues from data of TCGA.**

**Fig. S2. Kaplan-Meier overall survival and disease-free survival analysis for EZH2 using PRAD TCGA dataset.**

**Fig. S3. EZH2 and TGFBR2 expression were inversely correlated in PCa patients using PRAD TCGA dataset.**

**Fig. S4. Bar diagrams which represented the relative protein expression levels of TGFBR2 (A), HIF1A (B), and HIF2A (C) in normoxic or hypoxic condition.**

**Fig. S5. Bar diagrams which represented the relative protein expression levels of EZH2 (A), H3k27me3 (B), and TGFBR2 (C) after treating with EZH2 siRNAs or DNZep.**

**Fig. S6. Bar diagrams which represented the relative protein expression levels of EZH2 in normoxia or hypoxia.**

**Fig. S7. Western blot showed that siRNA decreased the protein expression level of HIF-1a, especially in DU145 and PC3 cells.**

**Fig. S8. Bar diagrams which represented the relative protein expression levels of TGFBR2 after miR-93 overexpression or downregulation.**

**Fig. S9. Bar diagrams which represented the colony numbers in different groups after miR-93 overexpression or downregulation.**

**Fig. S10. Bar diagrams which represented the relative mRNA expression levels of E-cadherin (A), N-cadherin (B), Vimentin (C), Zeb1 (D), and Zeb2 (E) after treating with miR-93 mimics or inhibitor.**

**Table S1. Clinicopathological characters in our cohort of 56 PCa patients.**

**Table S2. Primer list used in this study.**

**Fig. S1. TGFBR2 expression was significantly reduced in prostate cancer tissues from data of TCGA.**

**A,** Box plot showed reduced expression of TGFBR2 in PCa. **B,** Expression profiles of TGFBR2 in prostate cancer tissues and corresponding normal tissues. Pictures were generated by GEPIA (Gene Expression Profiling Interactive Analysis) (http://gepia.cancer-pku.cn/).


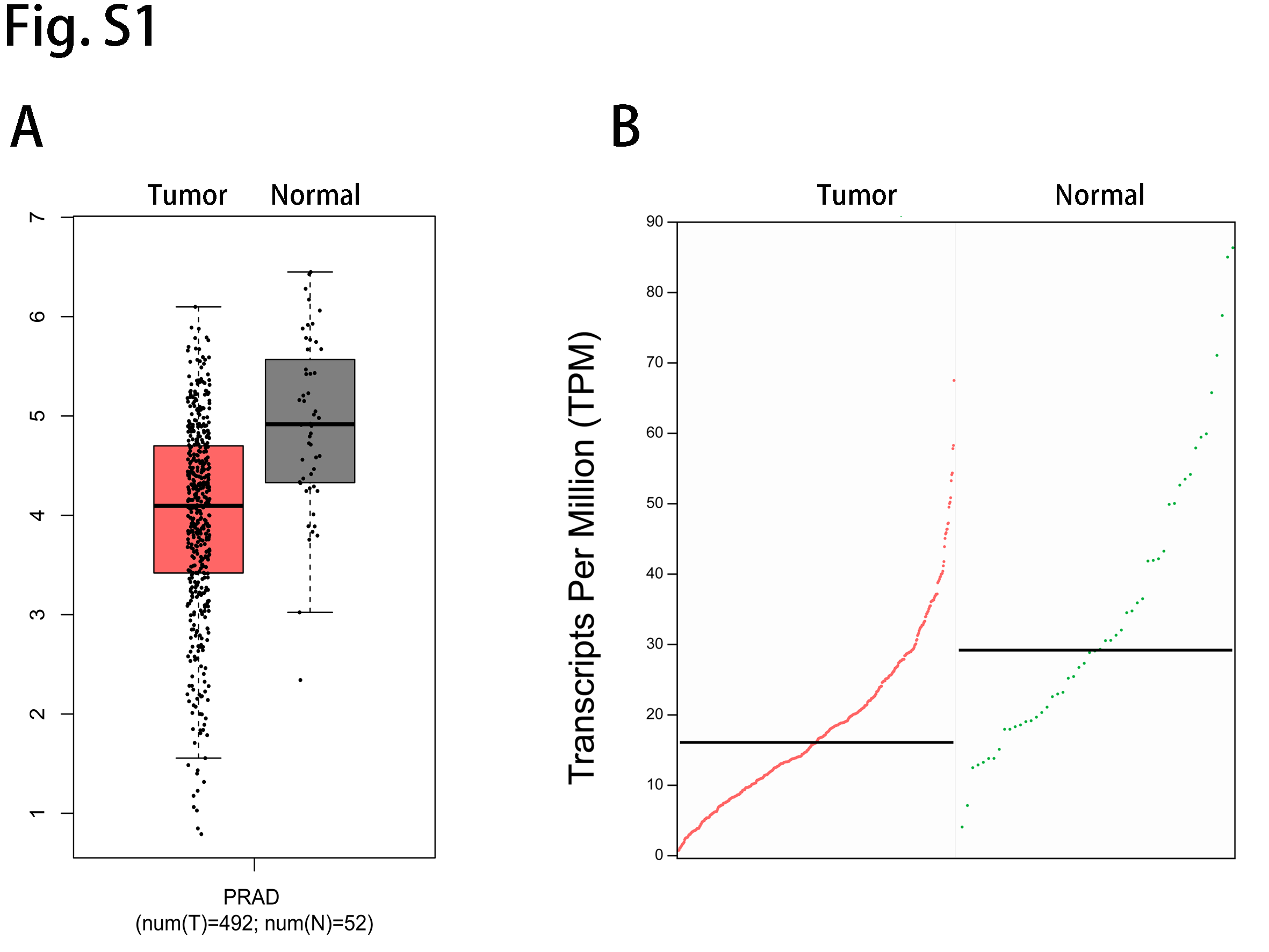


**Fig. S2. Kaplan-Meier overall survival and disease-free survival analysis for EZH2 using PRAD TCGA dataset.**

Kaplan-Meier curves with log-rank tests showed that patients with high expression levels of EZH2 had significantly shorter disease-free survival (HR=2.2, p < 0.001) and relative shorter overall survival (HR=4.1, p=0.054) than those with low EZH2 expression.


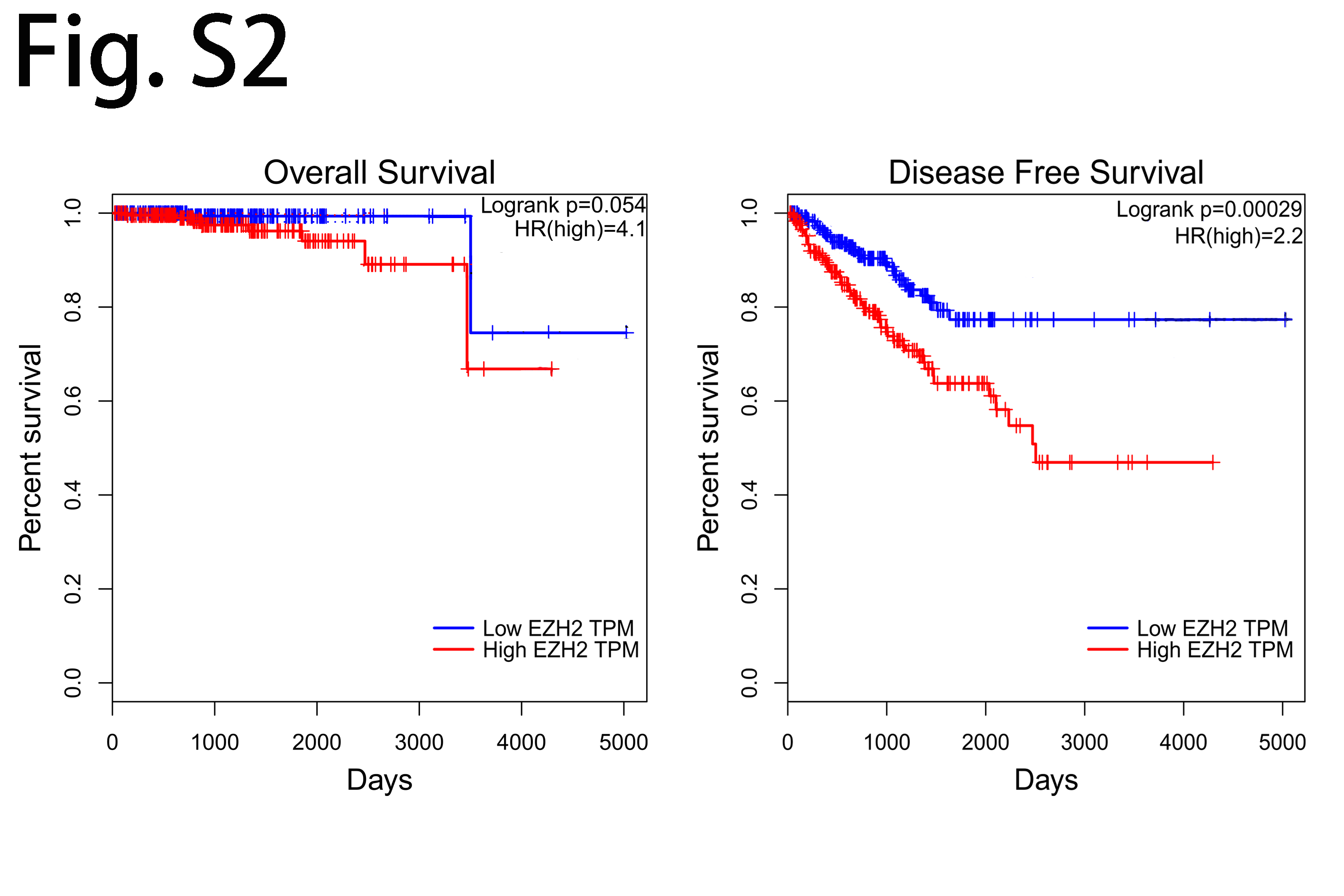


**Fig. S3. EZH2 and TGFBR2 expression were inversely correlated in PCa patients using PRAD TCGA dataset.**

Negative correlation between EZH2 and TGFBR2 expression levels in TCGA PRAD database (r=-0.3479, p<0.001).


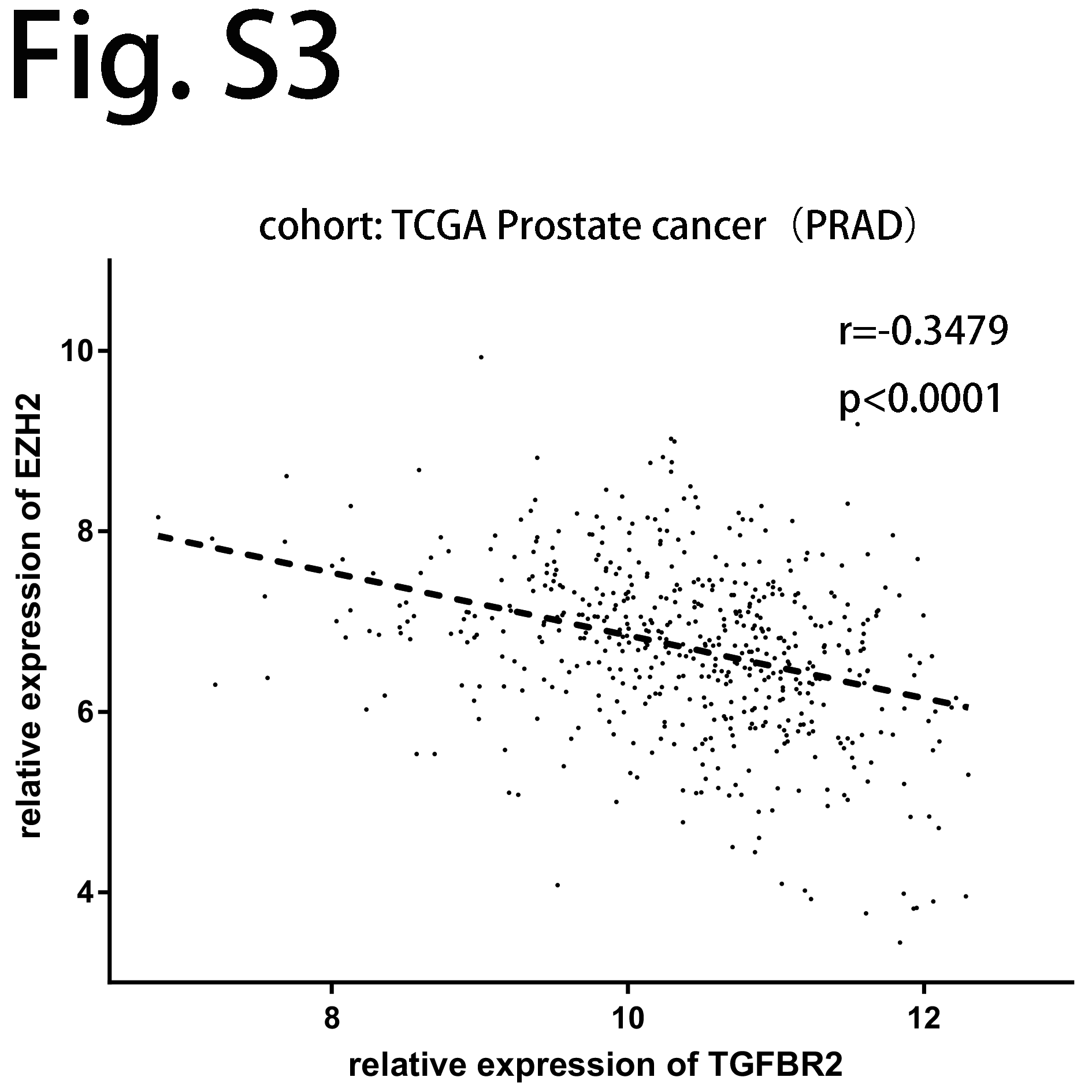


**Fig. S4. Bar diagrams which represented the relative protein expression levels of TGFBR2 (A), HIF1A (B), and HIF2A (C) in normoxic or hypoxic condition.**

Results were generated from the Western blot findings in Fig. 1A. The relative protein expression levels of aimed proteins were calculated as the ratio of grey values of aimed proteins and GAPDH.


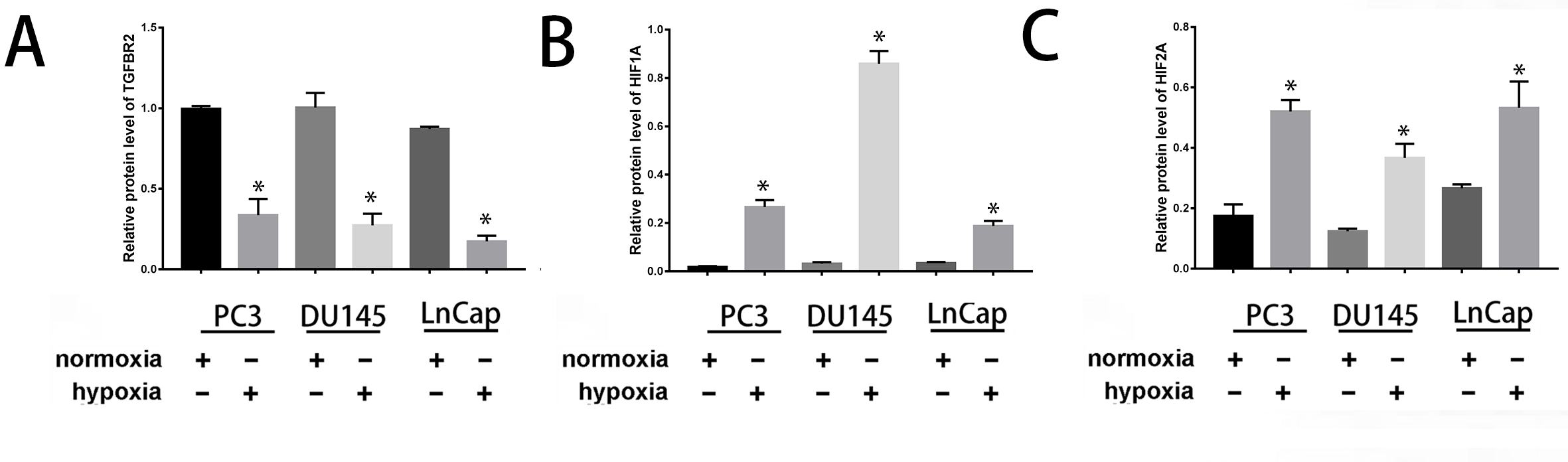


**Fig. S5. Bar diagrams which represented the relative protein expression levels of EZH2 (A), H3k27me3 (B), and TGFBR2 (C) after treating with EZH2 siRNAs or DNZep.**

Results were generated from the Western blot findings in Fig. 2F. The relative protein expression levels of aimed proteins were calculated as the ratio of grey values of aimed proteins and GAPDH.


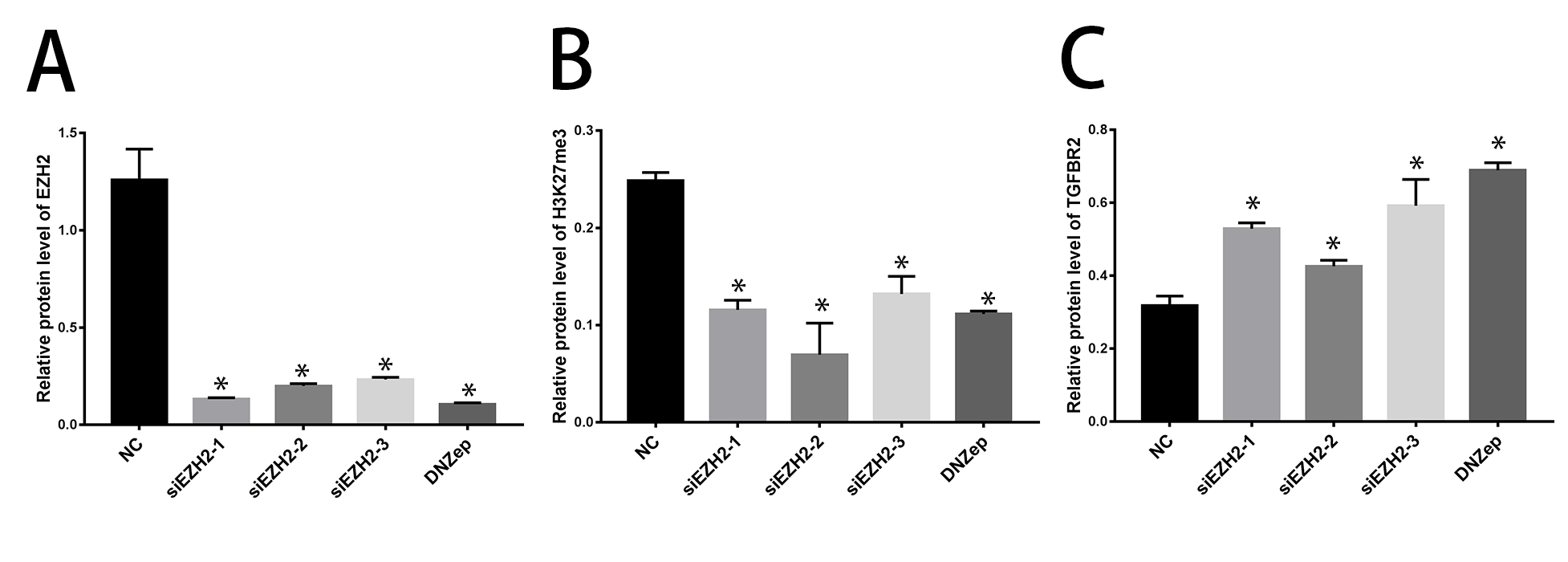


**Fig. S6. Bar diagrams which represented the relative protein expression levels of EZH2 in normoxia or hypoxia.**

Results were generated from the Western blot findings in Fig. 2I. The relative protein expression levels of aimed proteins were calculated as the ratio of grey values of EZH2 and GAPDH.


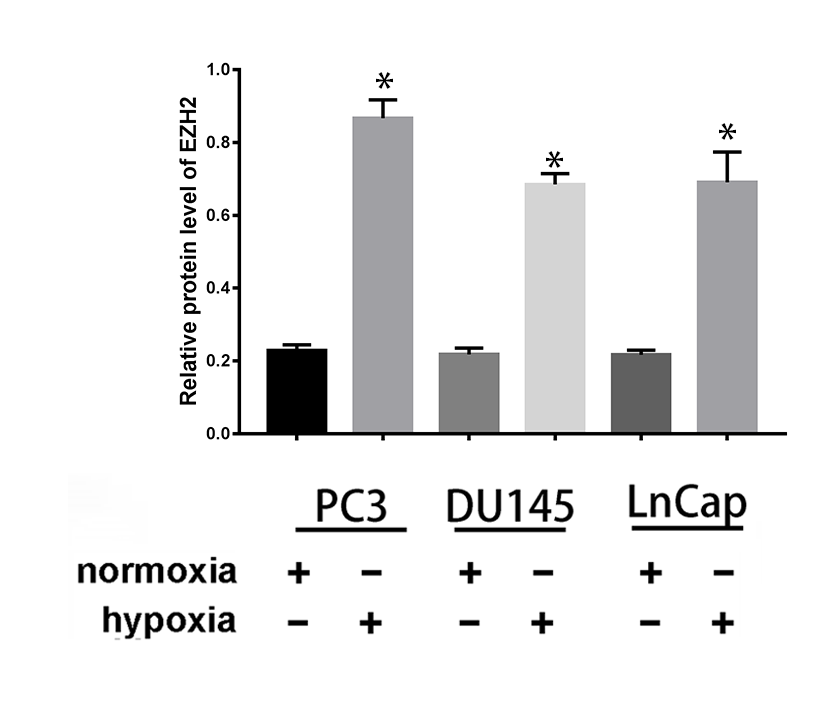


**Fig. S7. Western blot showed that siRNA decreased the protein expression level of HIF-1a, especially in DU145 and PC3 cells.**

HIF-1a silencing was effective in prostate cell lines such as DU145 and PC3, while no significant decrease was found in LnCap cell, as shown by western blot.


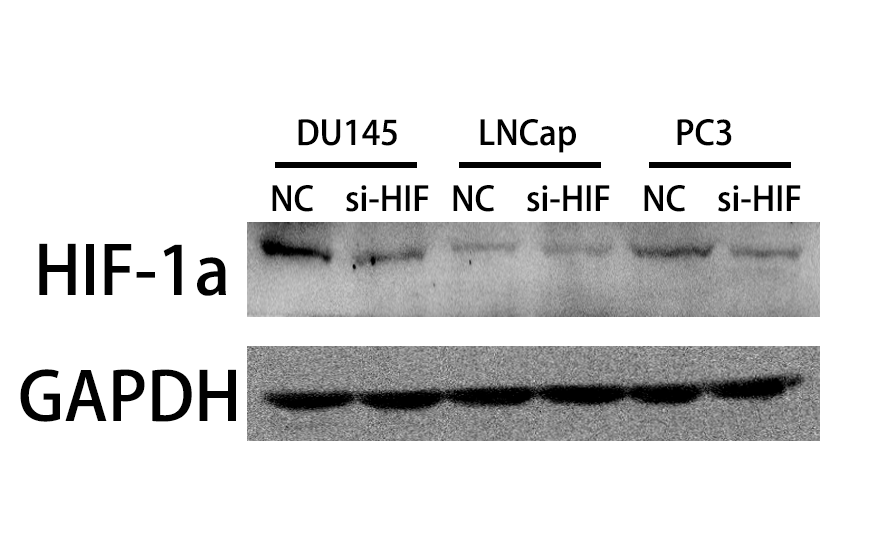


**Fig. S8. Bar diagrams which represented the relative protein expression levels of TGFBR2 after miR-93 overexpression or downregulation.**

Results were generated from the Western blot findings in Fig. 3I. The relative protein expression levels of aimed proteins were calculated as the ratio of grey values of aimed proteins and GAPDH.


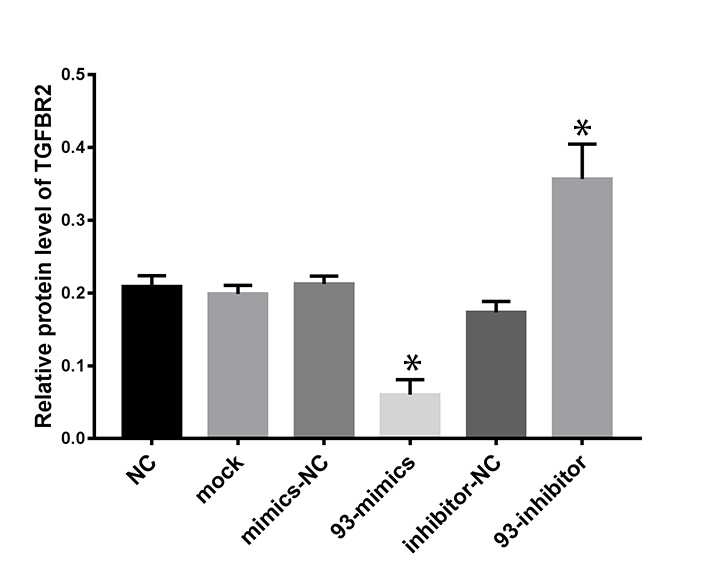


**Fig. S9. Bar diagram which represented the colony numbers in different groups after miR-93 overexpression or downregulation.**

Results were generated from the Colony formation assays in Fig. 4B. The colony numbers were calculated in different groups.


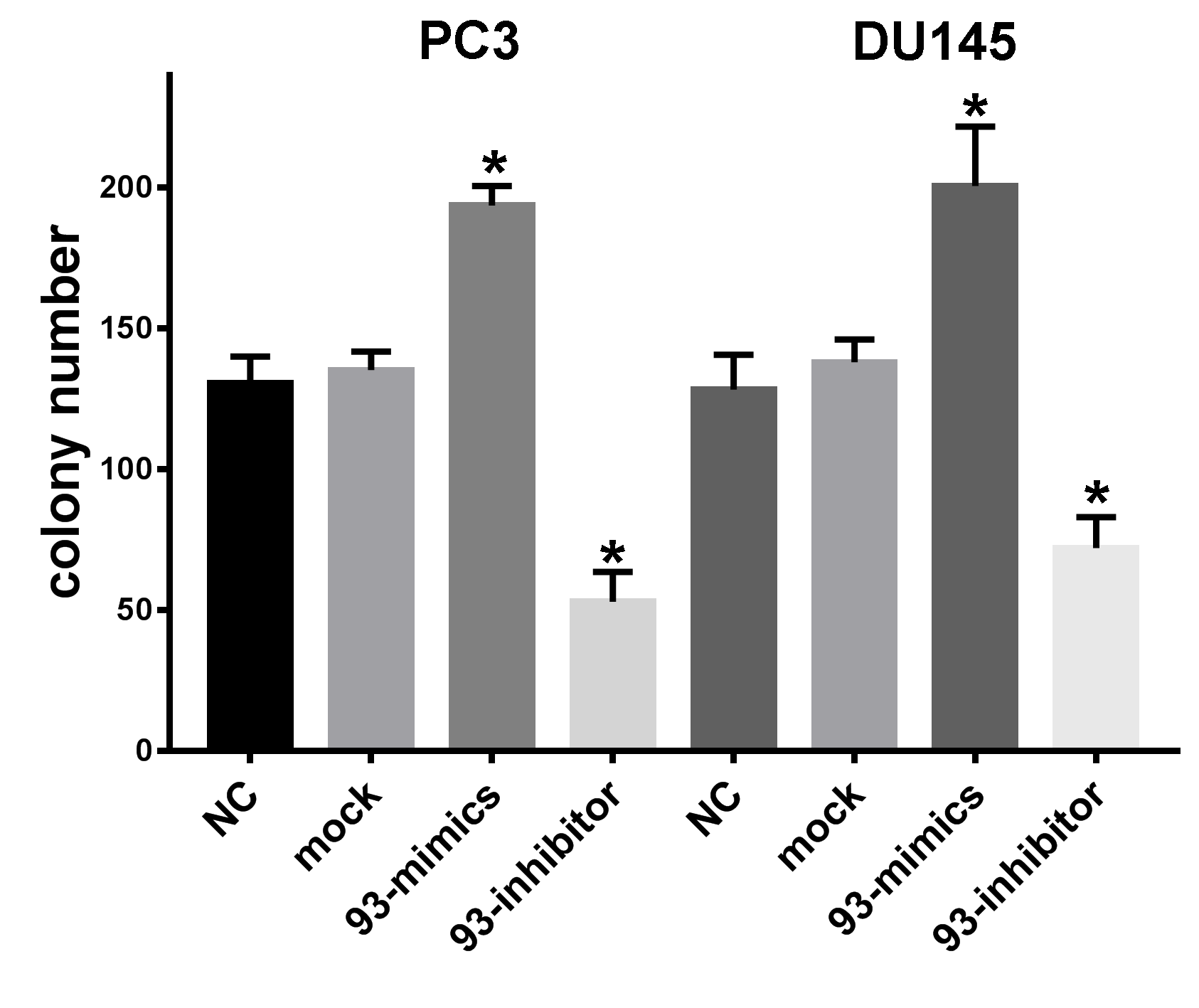


**Fig. S10. Bar diagrams which represented the relative mRNA expression levels of E-cadherin (A), N-cadherin (B), Vimentin (C), Zeb1 (D), and Zeb2 (E) after treating with miR-93 mimics or inhibitor.**

Results were generated from the RT-qPCR findings of EMT-related molecules. The relative mRNA expression levels of aimed molecules were calculated after defining the expression in negative control groups as 1.


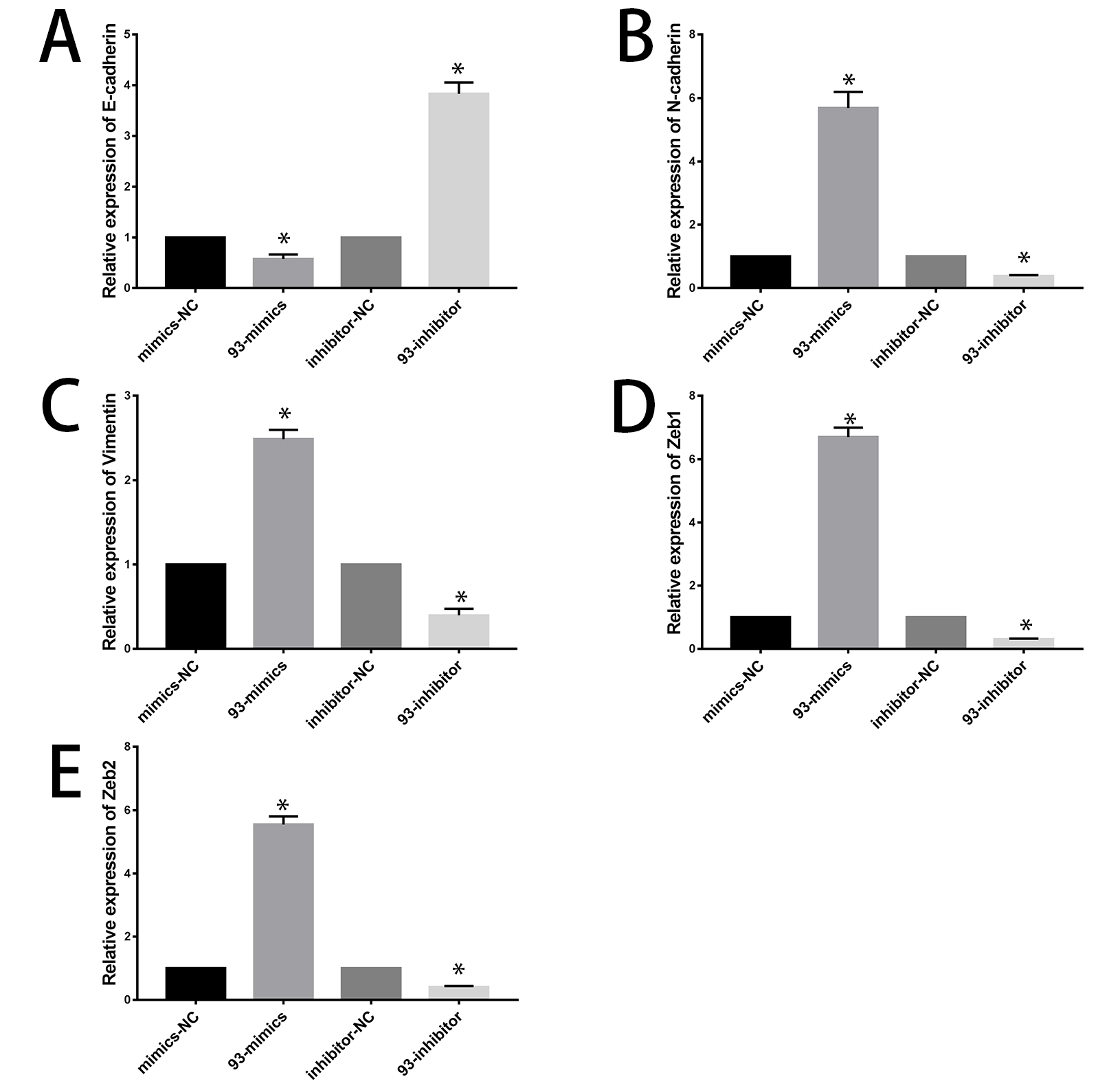


**Table S1.** Clinicopathological characters in our cohort of 56 PCa patients.

| Category | Value |
| --- | --- |
| No. patients | 56 |
| Age, median (range), yr | 65 (51-85) |
| BMI, mean ± SD, kg/m^2^ | 24.54 ± 3.12 |
| Tumor size, mean ± SD, cm | 2.05 ± 0.78 |
| PSA, median (range), ng/mL  Pathological stage, No. (%) | 32.75(3.89-345.78) |
| pT1 | 2 (3.57) |
| pT2a | 3 (5.36) |
| pT2b-T2c | 27 (48.21) |
| pT3 | 18 (31.03) |
| pT4 | 6 (10.71) |
| Gleason score |  |
| GS < 7 | 17 (30.36) |
| GS = 7 | 20 (35.71) |
| GS > 7 | 19 (33.93) |
| Local recurrence, No. (%) | 2 (3.57) |
| Metastasis, No. (%) | 6 (10.71) |
| BMI, body mass index; pT, pathological stage;  PSA, prostate specific antigen; GS, Gleason score. | |

**Table S2. Primer list used in this study.**

| Primer | Name | (5'-3') | SEQUENCE (5'-3') |
| --- | --- | --- | --- |
| qRT-PCR | TGFBR2 | Sense | ACTGCCCATCCACTGAGACAT |
|  |  | Antisense | CCATACAGCCACACAGACTTCC |
|  | EZH2 | Sense | CCGAGAGTGTGACCCTGACCT |
|  |  | Antisense | CTTTTTGGAGCCCCGCTGAAT |
|  | E-cadherin | Sense | GAGTGCCAACTGGACCATTCAGTA |
|  |  | Antisense | AGTCACCCACCTCTAAGGCCATC |
|  | N-cadherin | Sense | GATGTTGAGGTACAGAATCGT |
|  |  | Antisense | GGTCGGTCTGGATGGCGA |
|  | Vimentin | Sense | GGTGGACCAGCTAACCAACGA |
|  |  | Antisense | TCAAGGTCAAGACGTGCCAGA |
|  | Zeb1 | Sense | AAGAATTCACAGTGGAGAGAAGCCA |
|  |  | Antisense | CGTTTCTTGCAGTTTGGGCATT |
|  | Zeb2 | Sense | GGGGTACCATGCGAACTGCCATCTGA |
|  |  | Antisense | TTGCGGCCGCGTGCTTCAAAGAACAGGGTG |
|  | GAPDH | Sense | TGCACAGGAGCCAAGAGTGAA |
|  |  | Antisense | CACATCACAGCTCCCCACCA |
| MSP | TGFBR2 | Methylation forward | TTAGGAAATATGATTGGTAGTTACGA |
|  |  | Methylation reverse | CTATCCCGAACGAATACACG |
|  |  | Unmethylation forward | TTAGGAAATATGATTGGTAGTTATGA |
|  |  | Unmethylation reverse | ACTCCTATCCCAAACAAATACACAC |
| siRNAs | siTGFBR2-1 | Sense | GACCUCAAGAGCUCCAAUATT |
|  |  | Antisense | UAUUGGAGCUCUUGAGGUCTT |
|  | siTGFBR2-2 | Sense | CGACAUGAUAGUCACUGACTT |
|  |  | Antisense | GUCAGUGACUAUCAUGUCGTT |
|  | siEZH2-1 | Sense | GCUAGGUUAAUUGGGACCATT |
|  |  | Antisense | UGGUCCCAAUUAACCUAGCTT |
|  | siEZH2-2 | Sense | CCCAACAUAGAUGGACCAATT |
|  |  | Antisense | UUGGUCCAUCUAUGUUGGGTT |
|  | siEZH2-3 | Sense | CCAACACAAGUCAUCCCAUTT |
|  |  | Antisense | AUGGGAUGACUUGUGUUGGTT |
|  | siHIF1A-1 | Sense | GUGAUGAAAGAAUUACCGATT |
|  |  | Antisense | UCGGUAAUUCUUUCAUCACTT |
|  | siHIF1A-2 | Sense | CGGCGAAGUAAAGAAUCUGTT |
|  |  | Antisense | CAGAUUCUUUACUUCGCCGTT |
